# Generic model to unravel the deeper insights of viral infections: an empirical application of evolutionary graph coloring in computational network biology

**DOI:** 10.1186/s12859-024-05690-0

**Published:** 2024-02-16

**Authors:** Arnab Kole, Arup Kumar Bag, Anindya Jyoti Pal, Debashis De

**Affiliations:** 1 Department of Computer Application, The Heritage Academy, Kolkata, W.B. 700107 India; 2 Beckman Research Institute of City of Hope, Duarte, CA 91010 USA; 3 University of Burdwan, Burdwan, W.B. 713104 India; 4 Department of Computer Science and Engineering, Maulana Abul Kalam Azad University of Technology, Nadia, W.B. 741249 India

**Keywords:** Differential evolution, Protein–protein interaction networks, Drug target, Generic model, Hub proteins, KEGG pathway, Respiratory disorder

## Abstract

**Purpose:**

Graph coloring approach has emerged as a valuable problem-solving tool for both theoretical and practical aspects across various scientific disciplines, including biology. In this study, we demonstrate the graph coloring’s effectiveness in computational network biology, more precisely in analyzing protein–protein interaction (PPI) networks to gain insights about the viral infections and its consequences on human health. Accordingly, we propose a generic model that can highlight important hub proteins of virus-associated disease manifestations, changes in disease-associated biological pathways, potential drug targets and respective drugs. We test our model on SARS-CoV-2 infection, a highly transmissible virus responsible for the COVID-19 pandemic. The pandemic took significant human lives, causing severe respiratory illnesses and exhibiting various symptoms ranging from fever and cough to gastrointestinal, cardiac, renal, neurological, and other manifestations.

**Methods:**

To investigate the underlying mechanisms of SARS-CoV-2 infection-induced dysregulation of human pathobiology, we construct a two-level PPI network and employed a differential evolution-based graph coloring (DEGCP) algorithm to identify critical hub proteins that might serve as potential targets for resolving the associated issues. Initially, we concentrate on the direct human interactors of SARS-CoV-2 proteins to construct the first-level PPI network and subsequently applied the DEGCP algorithm to identify essential hub proteins within this network. We then build a second-level PPI network by incorporating the next-level human interactors of the first-level hub proteins and use the DEGCP algorithm to predict the second level of hub proteins.

**Results:**

We first identify the potential crucial hub proteins associated with SARS-CoV-2 infection at different levels. Through comprehensive analysis, we then investigate the cellular localization, interactions with other viral families, involvement in biological pathways and processes, functional attributes, gene regulation capabilities as transcription factors, and their associations with disease-associated symptoms of these identified hub proteins. Our findings highlight the significance of these hub proteins and their intricate connections with disease pathophysiology. Furthermore, we predict potential drug targets among the hub proteins and identify specific drugs that hold promise in preventing or treating SARS-CoV-2 infection and its consequences.

**Conclusion:**

Our generic model demonstrates the effectiveness of DEGCP algorithm in analyzing biological PPI networks, provides valuable insights into disease biology, and offers a basis for developing novel therapeutic strategies for other viral infections that may cause future pandemic.

**Supplementary Information:**

The online version contains supplementary material available at 10.1186/s12859-024-05690-0.

## Background

COVID-19, a global pandemic that emerged in late 2019, is caused by the highly transmissible severe acute respiratory syndrome coronavirus 2 (SARS-CoV-2). Belonging to the Coronaviridae family and Orthocoronavirinae subfamily, SARS-CoV-2 is a single-stranded positive-sense RNA (+ssRNA) virus, which belongs to Betacoronavirus genus among four classified coronavirus genera: Alphacoronavirus, Betacoronavirus, Gammacoronavirus, and Deltacoronavirus [1]. Within its genomic makeup, SARS-CoV-2 encodes sixteen non-structural proteins (Nsp1-16), four structural proteins (S, E, M, and N), and some accessory proteins (ORF) [2]. SARS-CoV-2-infected individuals commonly experience severe respiratory symptoms, such as fever, cough, and loss of taste and smell at the initial stage of infection. However, the impact of the virus extends beyond the respiratory system, resulting in additional manifestations such as nausea, intestinal obstruction, lung injury, and various complications affecting organs such as the kidney, gut, brain, heart, and other body parts [3, 4].

Till now, this global threat has taken almost seven-million human lives and infected more than seven hundred million people worldwide.^1^ During the pandemic period, numerous studies underscored the critical role of protein–protein interactions (PPIs) between SARS-CoV-2 virus proteins and human host factors in the initiation and progression of the infection, leading to the development of pathogenesis and associated human pathophysiological responses [5–9]. PPIs are the physical connections between two or more proteins. The collection of various protein–protein interactions forms a biological network called protein–protein interaction network (PPIN). Many researchers predicted these PPIs between SARS-CoV-2 virus and human proteins to point out the underlying mechanism of SARS-CoV-2 proteins-mediated disease propagation [5, 6, 10, 11]. The authors also predicted the affected pathways, target host proteins, associated disease progression, underlying cause of COVID-19 pathobiology, anti-viral drug repurposing and preventive therapeutic measures in a discrete way [6–8, 12]. But the actual host cellular targets of the SARS-CoV-2 virus, the precise pathways of virus transmission in humans, and the relative importance of each of those proteins and pathways are yet to be understood fully. Also, the long-term impacts of SARS-CoV-2 infection in humans still not fully explored. Furthermore, no generic model has been proposed yet that can reveal the underlying mechanism, transmission, effects, disease manifestations, and effective therapeutic strategies in a single framework for all viral infections. Therefore, it suggests an immense need to better understand viral infections and consequent dysregulation of human biological homeostasis in a comprehensive manner to offer new treatment or preventive strategies for COVID-19 like pandemic which may occur in future. Biological network establishment using virus-human protein–protein interactions, analysing the network for extrapolation to the next level of human–human protein–protein interaction network, identification of the crucial hub proteins at different levels in those biological networks along with bioinformatic analysis of those hub proteins to identify associated disease manifestations might have the potential to fulfill this purpose.

In Discrete Mathematics and Computer Science, any network is commonly represented using a graph. A graph is a collection of a set of vertices called nodes, and a set of links or connections between the nodes, called edges. Based on the direction of the edges or the weight associated with the edges, a graph can be directed, undirected, or weighted. Likewise, based on the functionalities and characteristics of the network, any biological network can be mapped mathematically to a directed, undirected, or weighted graph. Hence, biological protein–protein interaction network can also be mathematically represented as an undirected graph. In the mathematical representation of a PPI network, the proteins are represented as the vertices of the graph, and the interactions between the proteins are represented as the edges of the graph. Similarly, hub protein identification in a PPI network can also be mapped to the problem of vertex identification in a graph, which can be done in various ways, and most of them are successfully applied to numerous real-life combinatorial optimization problems which are NP-hard or NP-complete in nature [13, 14]. One of a very promising method to identify the vertices of a graph is graph coloring, which is also a well-known NP-complete combinatorial optimization problem [15]. Graph coloring is a fundamental concept in graph theory, involving the assignment of colors to the vertices (nodes) of a graph so that no two neighboring vertices, connected by an edge, share the same color. The least number of colors required to generate such coloring is known as the chromatic number of the graph. However, determining the chromatic number or finding an optimal coloring is considered an NP-complete problem, which is computationally challenging and time-consuming [16]. Consequently, meta-heuristic algorithms, including evolutionary algorithms and approximation methods, are commonly employed to find theoretical and practical solutions efficiently [17].

Graph coloring has wide applications in solving various optimization and allocation problems in real-world scenarios. Its successful implementation extends to diverse fields, including computational and network biology [18, 19]. Specifically, in biology, graph coloring has been proven helpful in modeling gene regulation networks, protein–protein interaction networks, disease-gene associations, drug target identification, functional annotation, disease subtyping, pathway analysis, and numerous other biological processes [20–24]. It is a straightforward and intuitive method that does not require complex algorithms or extensive computational resources and needs less memory consumption [22]. The approach is highly scalable and can be scaled to networks of varying sizes, making it suitable for small-scale and large-scale protein–protein interaction networks. As the network size increases, the graph coloring algorithm can efficiently handle the increased computational complexity without sacrificing accuracy [22, 25]. In addition, it is a flexible method that can be adapted to incorporate additional information or constraints, such as functional annotations or experimental data, to enhance the accuracy of hub protein identification [26].

**Fig. 1:**
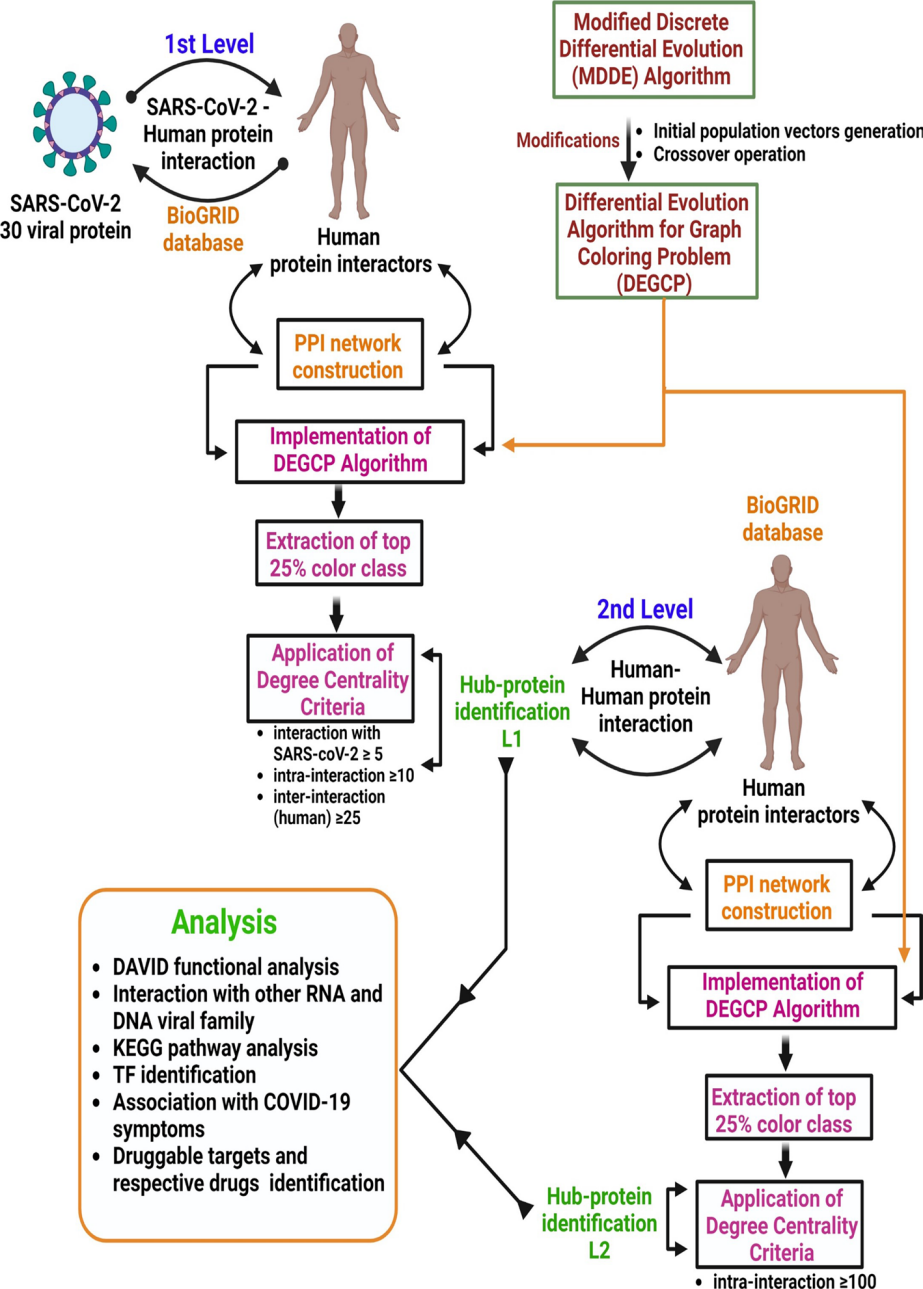
Schematic view of establishing SARS-CoV-2-human protein–protein interaction network and application of DEGCP algorithm to unravel the deeper insight of SARS-CoV-2 infection

In this study, we explore the application of an evolutionary graph coloring algorithm, namely DEGCP algorithm, in the context of a generic mathematical modelling of a biological protein–protein interaction network. The main objective is to propose a generic mathematical model for analysing a complex biological PPI network to uncover pivotal mediators associated with viral diseases and their effects on human pathophysiology. By utilizing DEGCP, we aim to analyze the complex PPI network efficiently and identify crucial hub proteins that play essential roles in the disease’s progression and manifestation. This investigation holds the potential to contribute valuable insights into the underlying biological mechanisms of viral diseases and may pave the way for novel therapeutic strategies in combating the disease and related conditions in future that may cause global threat. The proposed model (Fig. 1) consists of identifying pivotal hub proteins, their interactions with various DNA & RNA viral families, gene ontology and pathway analyses of those hub proteins, transcription factors identification, association of the hub proteins with various disease symptoms, and identification of their druggable targets and respective drugs. To validate our proposed model, we used the experimental data associated with COVID-19 disease, a pandemic that occurred all of the world in the recent past. The experimental outcomes aim that the proposed model can also be applied to other viral infections.

### Related work

Over the last few decades, literature surveys revealed various experiments that were conducted to analyze and predict the PPI for understanding different biological functions and identifying the crucial target proteins in protein–protein interaction networks [27–29]. An effective graph coloring-based integrative statistical algorithm has been designed for essential protein prediction [22]. The authors proposed hybridization methods consisting of graph coloring and artificial neural network (ANN) for finding the target proteins in infectious diseases [25, 26]. Graph theoretic approach was also proposed to find the infected pathways in viral disease [30]. PPIs between virus and host proteins were studied to highlight the underlying mechanism of SARS-CoV-2 proteins-mediated disease propagation [5]. A recent study exhaustively examined the molecular pathways, including pathways-based therapeutic targets for COVID-19 [6]. A comprehensive analysis was made using unsupervised machine learning method to highlight COVID-19-related affected pathways [7]. Bioinformatics analysis was conducted to understand the underlying molecular mechanism of advancement of SARS-CoV-2 infection by receiver operating characteristic (ROC) curve analysis [8]. Network topological analysis was performed to identify the potential target hub genes and affected pathways of COVID-19 [9]. SARS-CoV-2-induced pathways and corresponding drug repurposing strategies were identified by artificial neural network analysis using random walk with restart (RWR) method [12]. Computational identification of host genomic biomarkers influencing SARS-CoV-2 infections was made using statistical R-packages [31]. Underlying causes of COVID-19 pathobiology and prediction of potential therapeutic targets and effective drugs were examined extensively through virus-host protein interaction network study [32–35]. The authors attempted to develop combinatorial treatment strategies targeting both host factors and viral enzymes through comprehensive mapping of interactions between SARS-CoV-2 proteins and human proteins [36, 37]. An extensive computational investigation of the interactome of SARS-CoV-2 and human proteins was conducted for identifying possible virus-affected processes and potential protein binding sites [10]. The common pathways and molecular biomarkers in COVID-19 were identified through PPI network analysis which can cause pulmonary fibrosis and lung cancer [11].

### Motivation and contribution

Despite the enormous research, our understanding about the underlying mechanism and host targets of viral infections including SARS-CoV-2, effects of viral infections on host biological pathways, mechanism of development of disease pathology and its long-term impacts on hosts are still limited. The development of effective treatment options to prevent the disease and resolve associated manifestations are still an open challenge. Moreover, no single generic model exists till date that can address the issues associated with SARS-CoV-2 and other viral infections as well. These limitations motivated us to propose a new model that can enhance the deeper understanding of the mechanism of such viral infections and consequent pathophysiology. The understanding of viral-host interactions and their consequences is of utmost importance in comprehending the disease’s complex pathophysiology. Thus, in this study, we delve into the protein–protein interaction network of SARS-CoV-2 and human host proteins to identify crucial mediators that might shed light on the mechanisms underlying COVID-19 infection and its associated impact on human health. By elucidating these molecular interactions, we aim to contribute to the development of targeted therapeutic strategies and improve patient outcomes in this ongoing global health crisis, as well as for any other pandemic that can occur in future. We believe, our present research is a wise effort in this context.

Our proposed model, rooted in an evolutionary graph coloring algorithm, presents numerous advantages over traditional degree centrality criteria in discerning hub proteins within PPI networks. In contrast to degree centrality, which predominantly assesses the number of direct interactions a protein has, our model integrates the topological context of the entire network. It explores protein interactions comprehensively, encompassing both direct and indirect connections. The employed graph coloring methodology adheres to the principle that adjacent nodes (proteins) should not share the same color, signifying the absence of direct interactions. However, proteins with the same color may possess indirect connections through shared intermediates, indicating potential functional relationships. This approach recognizes that proteins involved in a pathway may contribute to the pathway’s activity without necessitating direct physical interactions. Degree centrality, reliant on the count of direct interactions, may be sensitive to the overall network size. This size of the networks or graphs keep on changing in realistic situation, and thus the networks or graphs are dynamic in nature. Any changes in network structure, such as node addition or removal, can significantly influence degree centrality and alter the identification of hub proteins. In contrast, our model demonstrates greater robustness to structural changes, adapting to alterations in network connectivity through the assigned colors reflecting local context connectivity, and thus is well-suited for dynamic graphs or dynamic network structures.

While our model does not entirely dismiss the significance of degree centrality, it incorporates it alongside additional weightings. The hub protein identification process involves two primary steps. Firstly, the PPI network graph undergoes chromatic coloring with minimal number of colors using a combination of differential evolution and sequential coloring algorithms. Differential evolution algorithm optimizes the ordering of the nodes so as to minimize the number of colors and the sequential algorithm assigns valid colors based on the degree and adjacency of the nodes. Subsequently, weights are assigned to human proteins based on color class, interactions with viral proteins, direct interactions with other proteins in the graph, and interactions with other human proteins outside the network. The incorporation of these diverse criteria, including but not limited to degree centrality, contributes to a more comprehensive hub protein identification process. The calculated Z-score, considering all aforementioned weightings, aids in establishing essential hub proteins based on a defined threshold. This multi-criteria approach enhances the probability of identifying crucial hub proteins compared to methods solely reliant on degree centrality criteria.

Our proposed work model demonstrates the applicability of graph coloring in computational network biology. We propose a generic mathematical model using graph coloring that can extract the potential human hub proteins associated with different viral infections. We have tested our models on SARS-CoV-2 infection occured in the recent past. It shows the importance of two levels of protein–protein interaction networks to understand the underlying mechanism and highlight the associated mediators of SARS-CoV-2 infection-induced disease manifestations. Furthermore, the experimental findings highlight the importance of hub proteins in COVID-19. It also conjectures the hub proteins associated biological pathways as the probable underlying mechanisms of SARS-Cov-2-mediated human pathophysiology. The bioinformatic analysis-based validation of our obtained results also identifies some essential transcription factors that might play an important role in altering biological signaling pathways. Finally, the proposed model also underscored the interconnection between SARS-CoV-2 infection and the long-term effects of the disease and accordingly highlighted some of the probable drug targets and respective drugs that might be beneficial to prevent the disease and resolve associated disorders. Furthermore, the experimental results also show that the proposed model can also be applied for other pandemic like COVID-19 that may occur in future.

## Materials and methods

### Mathematical formulation

Following are the notations used for the mathematical formulation of the proposed model. $V_p:$ set of virus proteins, $H_p:$ set of human proteins, $G=(V_G,E_G):$ an undirected graph *G*., $V_G:$ set of vertices, $E_G:$ set of edges whose elements are of the form $(v_i,v_j)$ where $v_i,v_j \in V_G$, $deg(v_i):$ degree of $v_i.$

$\chi (G):$ chromatic number of a graph *G*.

$C_i=\left\{ C_1,C_2,C_3,\cdots C_n\right\} :$ set of color classes.

$$C(v_i):$$ color of $$v_i.$$

$V_{IC}(x):$ number of virus protein interactors of *x*.

$TOTH_{IC}(x):$ total number of human protein interactors of *x*.

$INTERH_{IC}(x):$ number of inter human protein interactors of *x*.

$INTRAH_{IC}(x):$ number of intra human protein interactors of *x*.

*L*-1, *L*-2 graphs: $G=(V_G,E_G)$ where $V_G=\left\{ v_i:v_i \in H_p\right\} ,$ and $E_G=\left\{ (v_i,v_j)\right\}$ where $v_i,v_j\in H_p,$ $i \ne j,$ and $v_i$ is interacting with $v_j.$

$HUB_p:$ set of human hub proteins.

*Z*(*x*) :  Z-score of *x*., *X*(*x*) :  raw score of *x*., $\mu :$ mean, $\sigma :$ standard deviation.

For a given graph $G=(V_G,E_G)$, graph coloring problem can be mathematically defined as follows:

$$\begin{aligned} min \sum _{k=1}^{m}c_k \end{aligned}$$

1 $$\begin{aligned}{} & {} s.t.\; \; \; \sum _{k=1}^{m}u_{vk}=1 \end{aligned}$$

2 $$\begin{aligned}{} & {} u_{vk}+u_{wk} \le c_k \; \forall \;(v,w) \in E_G, \;k \in \left\{ 1,2,\cdots m\right\} \end{aligned}$$

3 $$\begin{aligned}{} & {} u_{vk},c_{k} \in \left\{ 0,1\right\} \; \forall \;v \in V_G, \;k \in \left\{ 1,2,\cdots m\right\} \end{aligned}$$

where *m* is the upper bound of the chromatic number $\chi (G)$, $c_k=1$ for all used color *k*, and $u_{vk}=1$ if $C(v)=k$ $\forall v \in V_G$. Equation (2) prevents two vertices to be assigned the same color values.

The hub human proteins are extracted at different levels based on the color values of the vertices of the graph and weightages given for different types of interactions. The first set of hub proteins at first-level are calculated as

4 $$\begin{aligned} HUB_p(L-1)=\left\{ v_i:v_i \in H_p,Z(v_i) \ge \theta \right\} \end{aligned}$$

where

5 $$\begin{aligned}{} & {} Z(v_i)=\frac{X(v_i)-\mu }{\sigma } \end{aligned}$$

6 $$\begin{aligned}{} & {} X(v_i)=X_1(v_i)+X_2(v_i)+X_3(v_i) \end{aligned}$$

7 $$\begin{aligned}{} & {} X_1(v_i)={\left\{ \begin{array}{ll} w_1 &{} \text { if } C(v_i)\leqslant \frac{n}{100}\cdot \chi (G) \; \; and \; \; V_{IC}(v_i)\geqslant \phi \\ \frac{w_1}{2} &{} \text { if } C(v_i)\leqslant \frac{n}{100}\cdot \chi (G) \; \; and \; \; V_{IC}(v_i)< \phi \\ \frac{w_1}{4} &{} \text { if } C(v_i)> \frac{n}{100}\cdot \chi (G) \; \; and \; \; V_{IC}(v_i)\geqslant \phi \\ -w_1 &{} \text { if } C(v_i)> \frac{n}{100}\cdot \chi (G) \; \; and \; \; V_{IC}(v_i)< \phi \end{array}\right. } \end{aligned}$$

8 $$\begin{aligned}{} & {} X_2(v_i)={\left\{ \begin{array}{ll} w_2 &{} \text { if } C(v_i)\leqslant \frac{n}{100}\cdot \chi (G) \; \; and \; \; INTRAH_{IC}(v_i)\geqslant \psi \\ \frac{w_2}{2} &{} \text { if } C(v_i)\leqslant \frac{n}{100}\cdot \chi (G) \; \; and \; \; INTRAH_{IC}(v_i)< \psi \\ \frac{w_2}{4} &{} \text { if } C(v_i)> \frac{n}{100}\cdot \chi (G) \; \; and \; \; INTRAH_{IC}(v_i)\geqslant \psi \\ -w_2 &{} \text { if } C(v_i)> \frac{n}{100}\cdot \chi (G) \; \; and \; \; INTRAH_{IC}(v_i)< \psi \end{array}\right. } \end{aligned}$$

9 $$\begin{aligned}{} & {} X_3(v_i)={\left\{ \begin{array}{ll} w_3 &{} \text { if } C(v_i)\leqslant \frac{n}{100}\cdot \chi (G) \; \; and \; \; INTERH_{IC}(v_i)\geqslant \eta \\ \frac{w_3}{2} &{} \text { if } C(v_i)\leqslant \frac{n}{100}\cdot \chi (G) \; \; and \; \; INTERH_{IC}(v_i)< \eta \\ \frac{w_3}{4} &{} \text { if } C(v_i)> \frac{n}{100}\cdot \chi (G) \; \; and \; \; INTERH_{IC}(v_i)\geqslant \eta \\ -w_3 &{} \text { if } C(v_i)> \frac{n}{100}\cdot \chi (G) \; \; and \; \; INTERH_{IC}(v_i)< \eta \end{array}\right. } \end{aligned}$$

where *n* is the percentages of color classes to be chosen, $X_1(v_i)$ is the score of the node calculated based on the the color classes and number of virus interactor proteins, $X_2(v_i)$ is the score of the node calculated based on the the color classes and number of intra-human interactor proteins, $X_3(v_i)$ is the score of the node calculated based on the the color classes and number of inter-human interactor proteins, and $w_1,w_2,w_3,\theta ,\phi ,\psi ,\eta$ are different threshold values.

The second set of hub proteins at second-level are calculated as

10 $$\begin{aligned} HUB_p(L-2)=\left\{ v_i:v_i \in H_p,Z(v_i) \ge \lambda \right\} \end{aligned}$$

where

11 $$\begin{aligned}{} & {} Z(v_i)=\frac{X_4(v_i)-\mu }{\sigma } \end{aligned}$$

12 $$\begin{aligned}{} & {} X_4(v_i)={\left\{ \begin{array}{ll} w_4 &{} \text { if } C(v_i)\leqslant \frac{m}{100}\cdot \chi (G) \; \; and \; \; INTERH_{IC}(v_i)\geqslant \zeta \\ \frac{w_4}{2} &{} \text { if } C(v_i)\leqslant \frac{m}{100}\cdot \chi (G) \; \; and \; \; INTERH_{IC}(v_i)< \zeta \\ \frac{w_4}{4} &{} \text { if } C(v_i)> \frac{m}{100}\cdot \chi (G) \; \; and \; \; INTERH_{IC}(v_i)\geqslant \zeta \\ -w_4 &{} \text { if } C(v_i)> \frac{m}{100}\cdot \chi (G) \; \; and \; \; INTERH_{IC}(v_i)< \zeta \end{array}\right. } \end{aligned}$$

where *m* is the percentage of color classes to be considered, $X_4(v_i)$ is the score of the node calculated based on the the color classes and number of inter-human interactor proteins, and $w_4,\lambda ,\zeta$ are different threshold values.

### Development of DEGCP algorithm

DEGCP algorithm (Algorithm 1) was developed based on the existing Modified Discrete Differential Evolution (MDDE) algorithm [38]. Two modifications were made in the existing MDDE algorithm. Firstly, we generated the initial populations with some guidance for producing promising vectors where the graph’s vertices were first sorted according to the degree in descending order. Then, the vertices of equal degree positions were interchanged randomly to generate different populations. Lastly, instead of a two-point crossover, the ordered crossover technique was incorporated in DEGCP algorithm.

**Algorithm 1:**
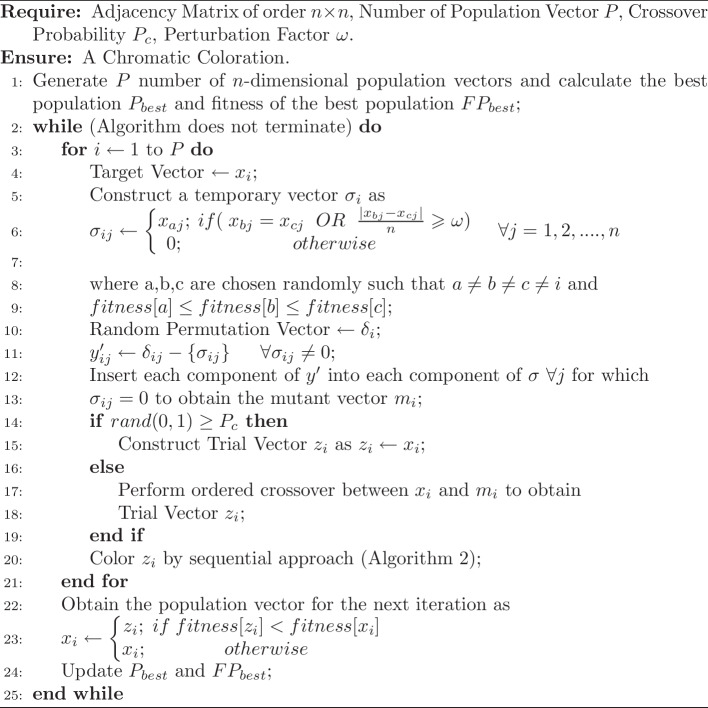
DEGCP Algorithm

**Algorithm 2:**
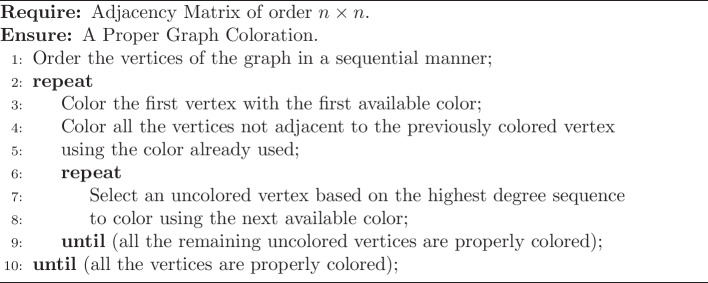
Sequential Coloring Algorithm

### First level human–human PPI network construction

Thirty SARS-CoV-2 virus proteins (E, M, N, S, Nsp1-Nsp-16, ORF10, ORF14, ORF3A, ORF3B, ORF6, ORF7A, ORF7B, ORF8, ORF9B, ORF9C) including spike, envelop and accessory proteins and their direct human interactors were extracted from BioGRID repository [39, 40]. The human interactors were included in our network contained all the experimentally identified physical interactors of virus proteins specified in the repository. Based on this criteria, 5225 unique human interactor proteins were selected for the first level network construction. The intra-interactions amongst these 5225 human proteins were extracted based on the same experimentally identified physical interactions criteria from BioGRID human–human protein interactome repository. Accordingly, 209,945 unique interactions were identified amongst 5225 human proteins which were further employed as the size and order respectively to create an undirected graph. This first-level of this PPI network was named PPIN1, and the undirected graph named L-1.

### Implementation of DEGCP algorithm and degree centrality criteria on L-1 graph

DEGCP algorithm was used to color the L-1 (5225, 209,945) graph with the parameters $P=50$, $\omega =0.6$, $P_c=0.75$, $Max\_Iteration=2000$. Consequently, a total of 60 disjoint color classes were identified. Then, we executed Eqs. (4)–(9) with different threshold values to extract first level potential human hub proteins. After several trails, we choose $n=25$, and the other threshold values as $\phi =5,\psi =10,\eta =25,w_1=5,w_2=1,w_3=1$, and $\theta =1$ to obtain a legitimate number of potential hub human proteins in the first level.

### Second level human–human PPI network construction

The second level of PPI network was constructed using the second level human protein interactors of 1082 L1 hub proteins. Accordingly, a total of 10,371 unique human interactors of L-1 hub proteins were extracted from BioGRID repository based on same experimentally identified physical interaction criteria. We then constructed a PPI network (PPIN2) and undirected graph L-2 based on 268,738 unique inter-interactions amongst 11,453 (10,371 $+$ 1082) human proteins.

#### Implementation of DEGCP algorithm and degree centrality criteria on L-2 graph

DEGCP algorithm was used similarly to color the L-2 (11,453, 268,738) graph with the parameters $P=50$, $\omega =0.6$, $P_c=0.75$, $Max\_Iteration=2000$. Consequently, a total of 80 disjoint color classes were identified. After that, we executed Eqs. (10)–(12) with $m=25$, and the different threshold values to extract second level potential human hub proteins. After several trails, we set the threshold values $\zeta =100,w_4=5$, and $\lambda =1$ to obtain the potential hub human proteins in the second level.

### Gene ontology analysis of the identified human hub proteins

Gene ontology (GO) analysis of the identified L-1 and L-2 hub proteins were performed using the DAVID Functional Annotation Tool [41, 42]. The Entrez Gene ID of the hub proteins were uploaded and the functional annotation were performed using DAVID. Biological Process (BP), Cellular Component (CC), and Molecular Function (MF) were extracted for the respective hub proteins. A modified Fisher Exact p-value, also known as the threshold of EASE Score, was used for gene enrichment analysis. Enrichment was performed by setting an EASE Score 0.01 and the minimum number of gene count as 5 for stringent gene enrichment.

### KEGG pathway analysis of the identified human hub proteins

Involvement of hub proteins in biological pathways were identified through KEGG Pathway Analysis using DAVID Functional Annotation Tool [41, 42]. The Entrez Gene ID of the hub proteins were uploaded and functional annotation were done by selecting the Pathways option. Only KEGG-PATHWAY was selected for specified pathway analysis. Like GO Analysis, the threshold value of the EASE Score for pathways analysis has also been set to 0.01, but unlike GO, the minimum number of gene count has been set to 15 to identify the most crucial pathways.

## Results

### Implementation of the DEGCP algorithm on undirected graph instances exhibits optimal/best-known results

To assess the applicability of the DEGCP algorithm to biological protein–protein interaction networks, first, we evaluated its performance on multiple undirected graph instances. Accordingly, this algorithm was tested on conventional DIMACS benchmark instances widely employed and originally suggested for graph coloring problems. The proposed DEGCP algorithm was tested fifty times independently on some of the different small, medium, and large-size graphs of varying complexity. Our experimental outcomes for respective graph instances with corresponding edges and vertices (columns 1–3, Table 1) demonstrated that the results produced by the proposed algorithm (column 6, Table 1) were the same as the optimal (column 4, Table 1) or best-known results (column 5, Table 1). Furthermore, DEGCP algorithm-derived results achieved optimal or best-known results with a 100% success rate for 17 graph instances (first 17 instances of column 8, Table 1), and the success rates for the other instances were within an acceptable range (60–80% for 18–20th instances of column 8, Table 1). Notably, our proposed algorithm produced the optimal results within an acceptable time limit (column 7, Table 1), which further suggested its efficiency and applicability for graphs with larger order and size. Altogether, the results demonstrated the effectiveness of the proposed algorithm on undirected graphs. Subsequently, we applied the DEGCP algorithm on SARS-CoV-2-Human protein–protein interaction network to delineate a deeper insight into viral infection and its consequences on human pathobiology.

**Table 1:** Results obtained by DEGCP Algorithm with time and success rate

DIMACS graph instances	Edges	Vertex	Known optimal $\chi (G)$	Best known result for $\chi (G)$ = ?	Results obtained by DEGCP	DEGCP time (S)	DEGCP success rate
homer	1629	561	13	13	13	0.12	50/50
fpsol2.i.1	11,654	496	65	65	65	0.59	50/50
inithx.i.1	18,707	864	54	54	54	1.64	50/50
zeroin.i.1	4100	211	49	49	49	0.11	50/50
le450_25a	8260	450	25	25	25	24.81	50/50
le450_25b	8263	450	25	25	25	8.28	50/50
myciel7	2360	191	8	8	8	0.02	50/50
1-Insertions_6	6337	607	?	7	7	0.16	50/50
2-Insertions_5	3936	597	?	6	6	0.12	50/50
3-Insertions_5	9695	1406	?	6	6	0.66	50/50
4-Insertions_4	1795	475	?	5	5	0.71	50/50
1-FullIns_5	3247	282	?	6	6	0.03	50/50
2-FullIns_5	12201	852	?	7	7	0.27	50/50
3-FullIns_5	33,751	2030	?	8	8	1.20	50/50
4-FullIns_5	77,305	4146	?	9	9	5.44	50/50
5-FullIns_4	11,395	1085	?	9	9	0.35	50/50
wap05a	43,081	905	?	50	50	118.31	50/50
school1	19,095	385	?	14	14	3145.21	30/50
school1_nsh	14,612	352	?	14	14	2459.35	35/50
will199GPIA	7065	701	?	7	7	924.26	40/50

### PPI network establishment using human interactors of SARS-CoV-2 and graph-coloring algorithm implementation predicts crucial first-level hub proteins

**Fig. 2:**
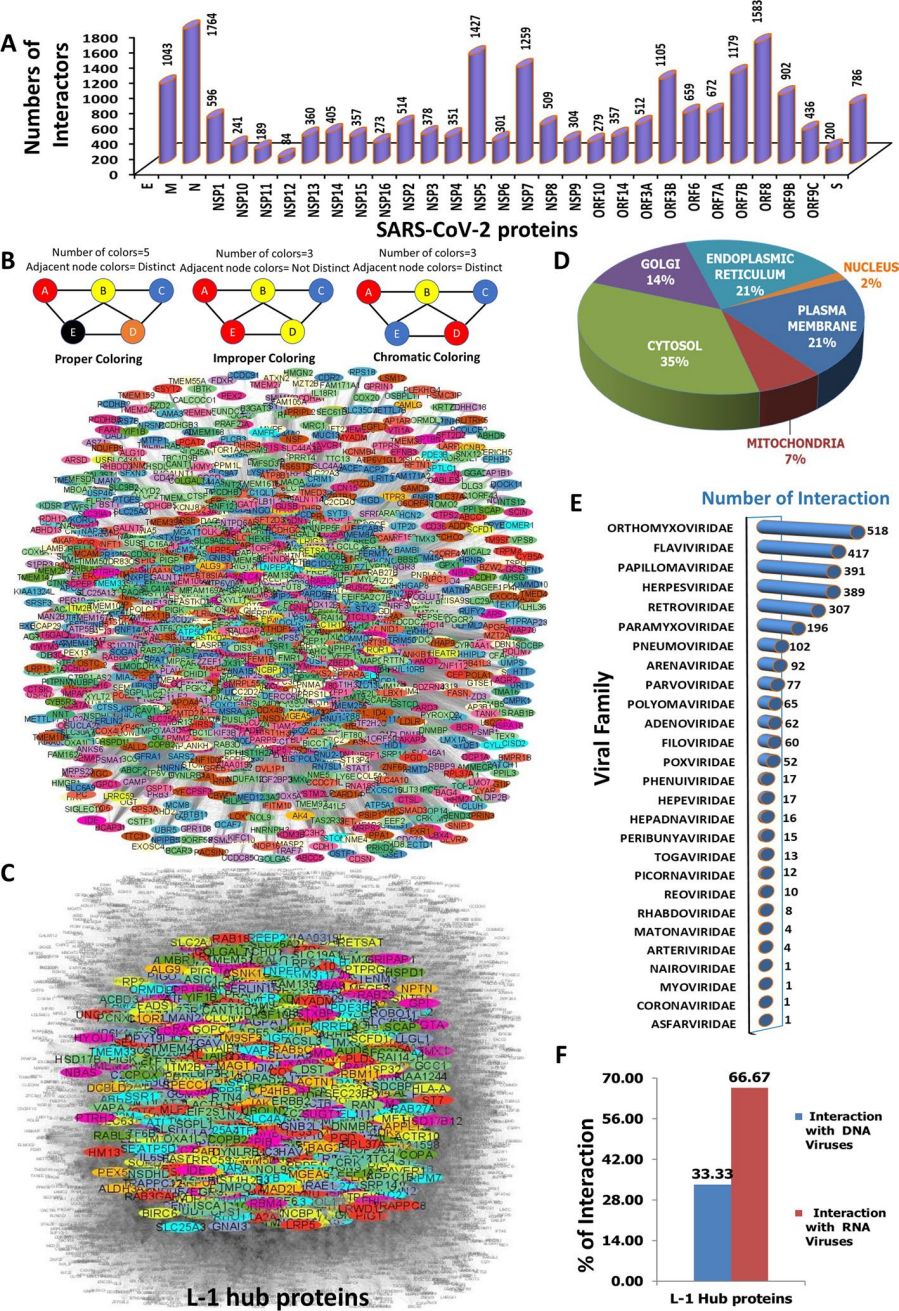
First-level protein–protein interaction network and graph coloring algorithm implemented filtration of HUB proteins. **A** Occurrence of human interactors of individual SARS-Cov-2 proteins. **B** PPI network of 5225 total interactors and visualization using chromatic graph coloring approach. **C** Highlighted PPI network of 1082 L-1 hub proteins and visualization using chromatic graph coloring approach. **D** Subcellular localization analysis of 1082 L-1 hub proteins. **E** Interaction frequency of 1082 L-1 hub proteins with 27 different groups of virus families. **F** Categorization of 27 interacting different groups of virus families based on genome content

To identify crucial human protein facilitators through which SARS-CoV-2 impacts the biological changes in infected individuals, we first constructed a protein–protein interaction network (PPIN). To create this PPIN, human interactors of thirty SARS-CoV-2 proteins were extracted from the BioGRID database (Fig. 2A). After that, the PPIN was constructed using 5225 human interactors of thirty SARS-CoV-2 proteins and their associated 2,09,945 interactions from the BioGRID human protein interactome database and visualized through a connected network (Fig. 2B). In this network, the human interactors of SARS-CoV-2 were denoted as different colored nodes based on the chromatic coloring approach of using minimum number (not necessarily optimum) of colors to designate adjacent connecting nodes with different colors. The edges represented direct interactions among these proteins, devoid of any indication of regulatory relationships, functional similarities, or directionality of regulation or signal or upstream-downstream demarcation. Thereafter, to find out the important hub proteins among those 5225 interactors, a graph coloring algorithm was implemented on PPIN and the first 25% of color classes were extracted. Additionally, weightage to the degree centrality of the nodes was added to extract potential hub proteins based on three criteria: interaction $\ge$ 5 with SARS-CoV-2 proteins, intra-interaction $\ge$ 10 within 5225 proteins, and inter-interaction $\ge$ 25 with next-level human interactors. The first criterion was used to add extra weightage based on the higher interaction probability with viral proteins, the second criterion was to add the additional impact of the viral interaction on the first-level interactors, and the third criterion was to add the higher likelihood of impacting on infected patients. It yielded 1082 potential hub proteins (Additional file 2: Table: Sheet S1) that were designated as L-1 hub proteinsand visualized through a network (Fig. 2C).

Evaluation of cellular localization of L-1 hub proteins showed 21% were plasma membrane, 21% were ER membrane, and 14% were golgi membrane-localized proteins whereas 35% were cytosolic proteins (Fig. 2D). It suggests that most of the essential direct interactors of SARS-CoV-2 were either membrane or cytosolic proteins. It also supports their higher possibility to interact with SARS-CoV-2 proteins which indirectly validates the potential of our graph coloring approach and weightage degree centrality-based hub protein identification method. The literature and HVIDB database [43] mapping of L-1 hub protein revealed that 80.4% (870 out of 1082) of them were previously reported as possible interactors of other than SARS-CoV-2 viruses (Additional file 2: Sheet S3), which belong to 27 different viral families (Fig. 2E). Notably, the hub proteins interacting with viral proteins from the same family may or may not fall into the same category. This is because of our undirected protein–protein interaction networking model, where color codes are not assigned based on functional similarity. As SARS-CoV-2 is an RNA virus family member, categorization based on the genetic content of interacting virus families indicates 33.33% of them were DNA virus families, whereas 66.67% were RNA virus families (Fig. 2F). It is noteworthy that the majority of the identified hub proteins, associated with both RNA and DNA viruses, may contribute to the manifestations of viral diseases. However, one-third of the DNA-virus family interactors may have distinctive roles in the context of SARS-COVID-19 responses. The complexity and distinct manifestations of COVID-19 compared to other viral diseases prompt the consideration that these hub proteins might play unique roles in the development of the complex disease associated with SARS-CoV-2. Alternatively, these hub proteins may act as common mediators for both DNA and RNA viral infections. This further suggests our method identified essential hub proteins with a higher probability of interacting with RNA viruses.

### PPI network construction of the second-level interactors of the first-level hub proteins and application of graph coloring algorithm predicts the important second-level hub proteins

**Fig. 3:**
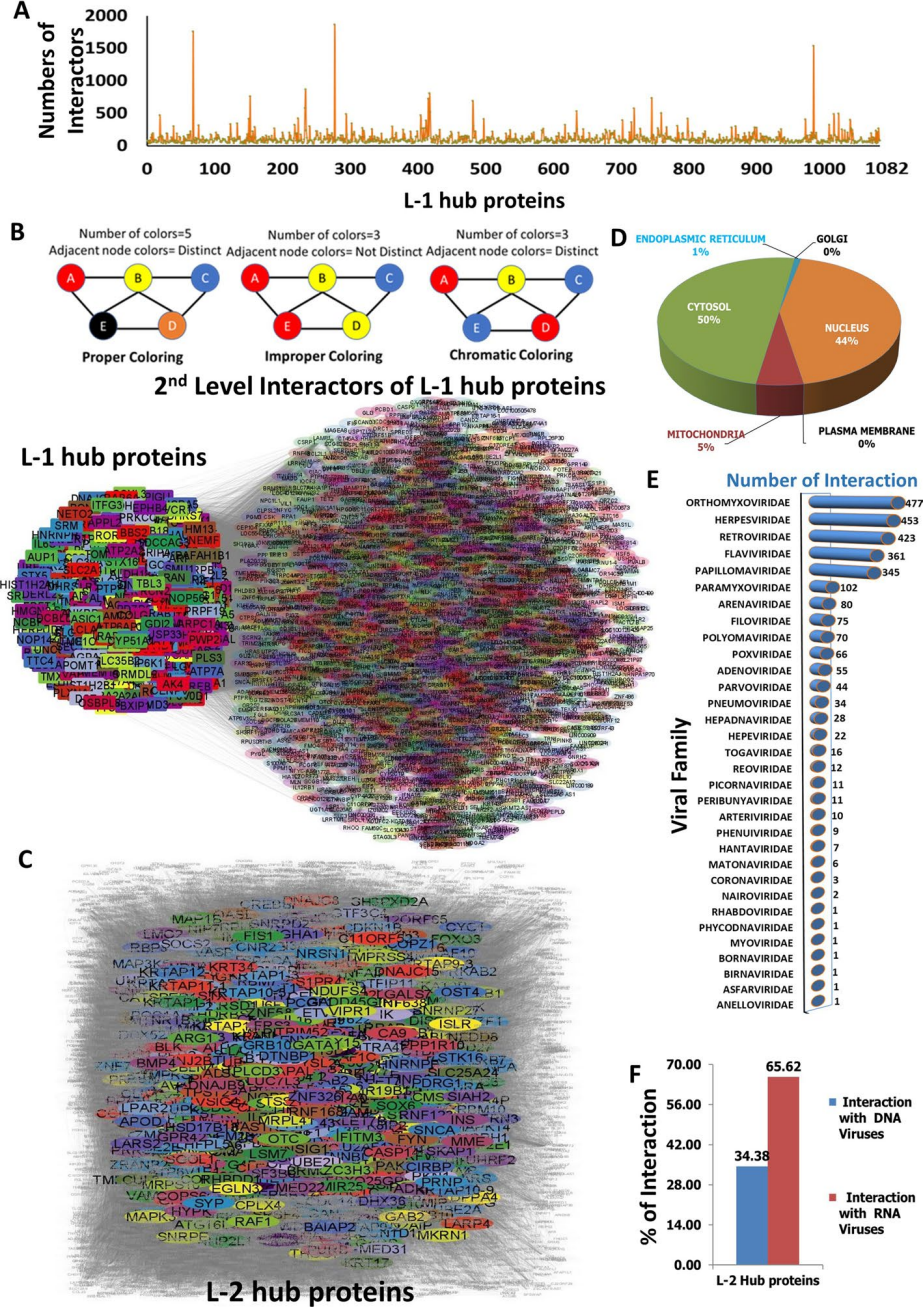
Second-level human–human protein–protein interaction network and graph coloring algorithm implemented filtration of L-2 hub proteins. **A** Frequency of human interactors of individual L-1 hub proteins. **B** PPI network of 1082 L-1 hub proteins and 10,371 total interactors highlighting L-1 hub proteins. **C** Highlighted PPI network of 1922 L-2 hub proteins and visualization. **D** Subcellular localization analysis of 1922 L-2 hub proteins. **E** Interaction frequency of 1922 L-2 hub proteins with 32 different groups of virus families. **F** Categorization of 32 interacting different groups of virus families based on DNA or RNA genome content

To characterize the second level of important human proteins through which L-1 hub proteins influence the biological alterations in infected individuals, a second protein–protein interaction network (PPIN2) was constructed. To generate the PPIN2 network the second-level interactors of 1082 L1-hub proteins were extracted from the BioGRID database (Fig. 3A). Subsequently, PPIN2 was created using 1082 L1-hub proteins and their second-level 2,68,738 unique inter-interactions with the rest of the 10,371 human interactors from the BioGRID human protein interactome database and presented through a network highlighting the L-1 proteins (Fig. 3B). Similar to PPIN1 network, nodes were colored based on the chromatic coloring approach and the edges denoted as direct interactions among the nodes, devoid of any indication of regulatory relationships, functional similarities, or directionality of regulation or signal or upstream-downstream demarcation. The chromatic coloring of nodes were applied based on the similar criteria of PPIN and the first 25% of color classes were extracted as the significant nodes. Furthermore, weightage to the degree centrality of the nodes was added to extract most potential hub proteins based on the criteria: intra-interaction $\ge$ 100 within a total of 11,453 interactors. This criterion was added to filter out the second-level hub proteins with a higher possibility of impacting infected patients. It yielded 1922 potential hub proteins (Additional file 2: Sheet S4) that were designated as L-2 hub proteins and highlighted in the total network (Fig. 3C).

Cellular localization analysis of L-2 hub proteins corresponds to 44% of nucleus-localized proteins and 50% of Cytosolic proteins and no or minimal plasma membrane, ER, or Golgi membrane proteins (Fig. 3D). It suggested the possibility that the majority of second-level interactors might be directly or indirectly involved in gene regulation which is also expected from the second-level interactors. Hence, it also validates the potential of our method to extract the important hub proteins. HVIDB database [43] mapping for L-2 hub proteins for the interaction information with other viruses revealed that 59% (1134 out of 1982) of them were previously reported as possible interactors for non-SARS-CoV-2 viruses (Additional file 2: Sheet S6). Grouping of these viruses according to different families showed they belonged to 32 different viral families (Fig. 3E). Similar to L-1 hub proteins, the identified L-2 hub proteins interacting with viral proteins from the same family may or may not fall into the same category because of our proposed protein–protein interaction networking structure. Additionally, genetic content-based categorization of these 32 virus families appeared as 34.38% of them were DNA virus families whereas 65.62% were RNA virus families (Fig. 3F). As explained earlier, one-third of the DNA-virus family interactors may have distinctive roles in the context of SARS-COVID-19 responses and might play unique roles in the development of the complex disease associated with SARS-CoV-2. This further suggests our method was able to find out important hub proteins which have a higher probability to interact with RNA viruses like SARS-CoV-2.

### Collective PPI network-graph coloring model identifies important biological and functional consequences of SARS-CoV-2 infection

**Fig. 4:**
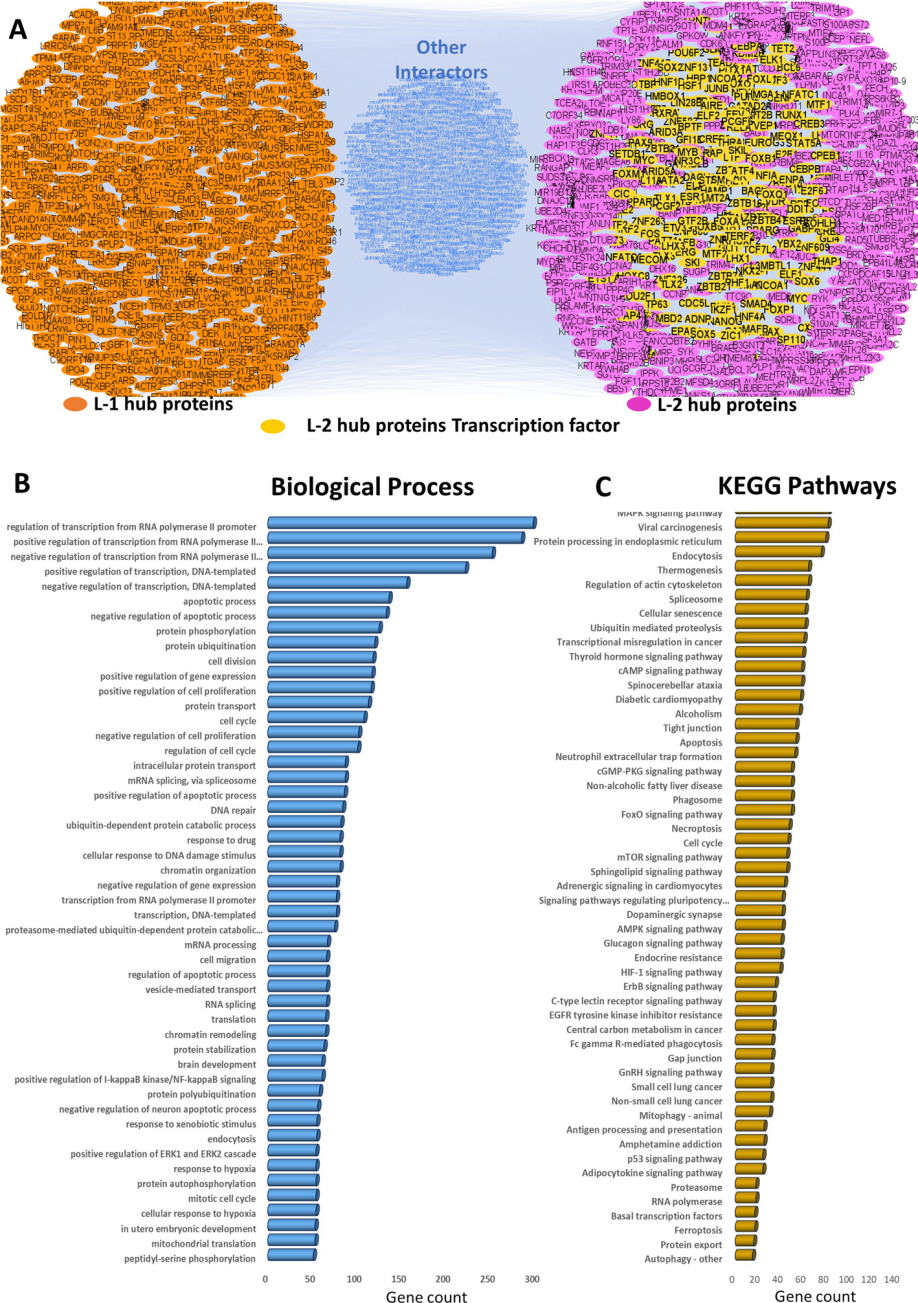
Biological and functional consequences of PPI network-Graph coloring model identified L-1 and L-2 hub proteins. **A** L-1 and L-2 hub proteins highlighted second level PPI network and identified transcription factors (TF) amongst those hub proteins after TF database mapping. **B** L-1 and L-2 proteins associated 50 biological processes and their gene count in each process. **C** KEGG pathway enrichment analysis of L-1 and L-2 hub proteins and 55 represented pathways and their gene count in each pathway

To demonstrate the efficiency of our proposed model for identifying SARS-CoV-2 infection-linked important hub proteins and to establish their biological implications, L-1 and L-2 hub proteins associated biological and functional consequences and their potentiality to alter the human gene expression in favor of them were explored. Therefore, transcription factor database [44] mapping and DAVID functional annotation tool-based analysis of L-1 and L-2 hub proteins were performed. Cellular localization analysis of L-1 and L-2 hub proteins (Fig. 2D, Additional file 2: Sheet S2, Fig. 3D, Additional file 2: Sheet S5) showed that the majority of L-1 hub proteins were cytosolic whereas most of the L-2 hub proteins were nuclear and cytosolic. In an extension of those findings, transcription factor (TF) database mapping on this combined PPI network of L-1 and L-2 proteins demonstrates that none of the L-1 hub proteins were TF whereas 206 out of 1,922 L-2 hub proteins were TF (Fig. 4A, Additional file 2: Sheet S9). Collectively this result substantiates the possibility that SARS-CoV-2 proteins interact with key first level interactor (L-1 hub proteins) which further interacts with the vital second-level proteins (L-2 hub proteins), which have the potential to enter inside the nucleus and alter gene expression to establish the disease manifestations. Furthermore, it signifies the potential of our proposed model to identify the crucial proteins related to SARS-CoV-2 infection to develop COVID-19 disease.

To further investigate the all-inclusive involvement of L-1 and L-2 hub proteins in the disease manifestation of COVID-19, DAVID functional analysis for analyzing their involvement in biological process were performed (Fig. 4B). The result revealed that a good number of of L-1 and L-2 hub proteins identified by our proposed model were associated with important biological process like regulation of transcription positively and negatively through the alteration of RNA-Pol-II or DNA template or regulating the mRNA splicing and processing as well as protein translation, which might be the possible ways of alteration of host gene expression in favor of the SARS-CoV-2 infection and disease manifestation. Also, a good number of proteins were involved in regulation of apoptosis, cell division and cell cycle, cell proliferation and cell migration, chromatin organization process which might be the possible ways SARS-CoV-2 alter the host cell fates towards the death to induce the injury in different organs mainly in the first infected sight, lung. Some of those identified L-1 and L-2 proteins were engaged in protein phosphorylation and their transport in different organellar location and degradation through ubiquitin pathway that might be the possible ways through which SARS-CoV-2 alters the hosts’ cellular signaling to support their favorable conditions. Our result suggested that another group of proteins were involved in endocytosis and more importantly in accordance to response in hypoxic conditions which might be the important one to support their survival and activity in less oxygen environment which is the common manifestation of SARS-CoV-2 infected hosts (Fig. 4B, Additional file 2: Sheet S7).

Furthermore, to delineate the L-1 and L-2 hub proteins’ involvement in biological pathways through which SARS-CoV-2 essentially alter the homeostasis of host cellular pathways, the KEGG pathways enrichment analysis was performed (Fig. 4C). The result showed that a large number of proteins were involved in important cellular signaling pathways like MAPK signaling, cAMP signaling, cGMP-PGK, FoxO and ErbB signaling pathways which might be responsible for altering the host cellular functions to support the infection and disease progression (Fig. 4C). A good number of hub proteins were involved in HIF-1 signaling pathways responsible to alter the pathways related to hypoxic condition, suggest that through this alteration SARS-CoV-2 might support their progression at less oxygen conditions. Likewise, proteins involved in p53 signaling pathway were the possible moderator of SARS-CoV-2 through which virus change the host cell death or cell proliferation of the infected organs. Similarly, large number of hub proteins were involved in different cancer related signaling pathways including lung cancer, suggested that those are responsible to change the large number of cellular pathways in favor of infection establishment and disease manifestations (Fig. 4C, Additional file 2: Sheet S8).

### This Proposed model underscores the connections between SARS-CoV-2 infection and its patho-physiology

To investigate the biological connection between SARS-CoV-2 infection and the development of its pathophysiology, the association of the L-1 and L-2 hub proteins with NIH defined COVID-19 manifestations were evaluated. Therefore, well established COVID-19 symptoms or pathophysiology as per NIH report were listed from the published literature search [45, 46]. The four major symptoms categories were identified as most established manifestations of COVID-19 as respiratory, cardiovascular, hematologic, and neuropsychiatric disorders. Based on the literature search (References given in the Additional file 1), the proteins associated with or involved for the development of those symptoms were extracted and categorized per group, as given in Table 2.

**Table 2:** Proteins involved in various cardiovascular, hematologic, neuropsychiatric and hematologic disorders

Category	Category protein count	Disease	Disease protein count
Cardiovascular	480	Arrhythmias	237
		Congestive Heart Failure	229
		Ischemic Heart Disease	24
		Myocardial Fibrosis	243
		Post Viral Myocarditis	149
Hematologic	1456	Deep Vein Thrombosis	1456
		Lung Fibrosis	1864
Respiratory	1938	Post Bacterial Pneumonia	211
		Pulmonary Embolism	96
		Anxiety	63
		Cerebral Vein Thrombosis	1424
		Depression	547
Neuropsychiatric	3309	Insomnia	235
		Post Traumatic Stress Disorder	778
		Seizures	431
		Stroke	117

**Fig. 5:**
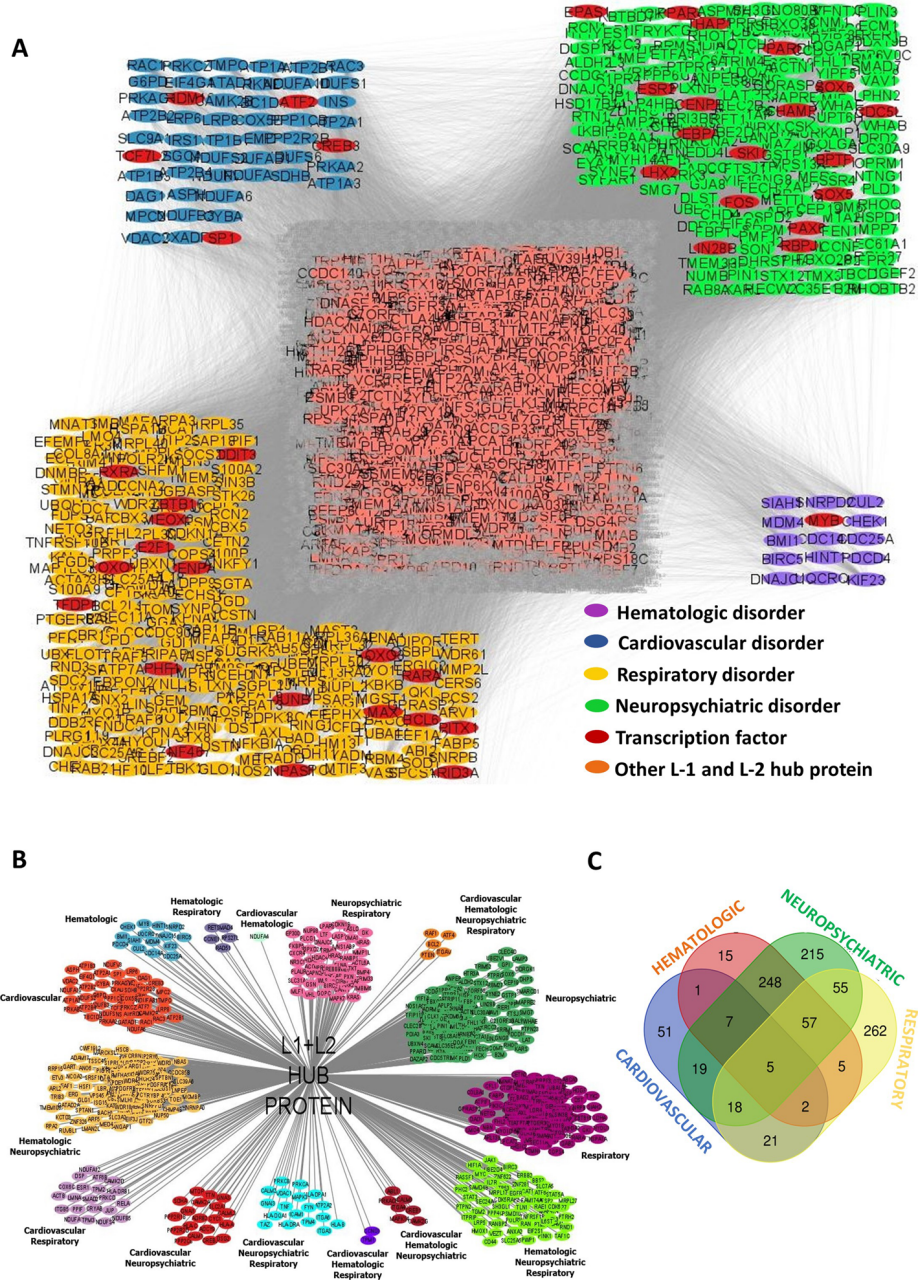
Connections of L-1 and L-2 hub proteins with SARS-CoV-2 infection and their association with pathophysiology. **A** Involvement and possible connections of highlighted L-1 and L-2 hub proteins with COVID-19 pathophysiology of respiratory, cardiovascular, hematologic, and neuropsychiatric disorders. **B** Detailed sub categorization of pathophysiology associated L-1 and L-2 proteins. **C** Venn diagram of distribution of L-1 & L-2 genes /proteins associated with pathophysiology of respiratory, cardiovascular, hematologic, and neuropsychiatric disorders

The extracted protein list was mapped on the L-1 and L-2 hub proteins-highlighted 2nd level of network and scrutinized the number of L-1/L-2 hub proteins involved in different symptoms. Accordingly, the result revealed that 262, 51, 15, and 215 number of hub proteins were associated respiratory, cardiovascular, hematologic, and neuropsychiatric disorders respectively (Fig. 5A, Additional file 2: Sheet S10). This result emphasized the importance of combined PPI-graph coloring model to find out important mediators or biological molecule behind the COVID-19 disease progression and its manifestations which can be targeted to resolve the disease or inhibit the development of disease pathophysiology. Further TF database mapping on the four major pathophysiology associated proteins groups showed that 18, 5, 1, and 18 number of TFs were found involved exclusively in respiratory, cardiovascular, hematologic and neuropsychiatric disorders, respectively. The details of such protein are given in Table 3. As TFs can modulate the expression of multiple genes together, so it further strengthens the possibilities that pathophysiology associated TFs might be the important for SARS-CoV-2 to alter the host biology simultaneously targets to resolve the condition. Further in-depth analysis of pathophysiology associated proteins revealed that there are some proteins which are solely associated with one pathophysiology, but a good number of proteins were associated with multiple conditions (Fig. 5B for name of those proteins, Fig. 5C for the count of proteins). These findings suggest that both group of proteins have distinct importance for the development of disease manifestations as well as in targeting them to neutralize the pathologic conditions. The first group of proteins might be important through which SARS-CoV-2 develop the particular organ specific pathophysiology and targeting to inhibit the information flow can resolve that particular condition. Whereas the second group of proteins might be important through which SARS-CoV-2 develop the disease pathophysiology in multiple organ and targeting to inhibit their function can resolve the complications of multiple organs together. Another possibility could be that the second group of proteins might be involved at the later stage of the SARS-CoV-2 infection when the complications were not confined to a particular organ. More specific studies required to confirm that possibilities and if so then those proteins could be targeted for the late stage of the COVID-19 patients to resolve the complications.

**Table 3:** Hub transcription factor proteins involved in combination of four disorder catagory

Type of disorder	Related Hub TF protein count	Related Hub TF protein name
Cardiovascular	5	ATF2, CREB3, PRDM16, SP1, TCF7L2
Hematologic	1	MYB
		BPTF, CDC5L, CEBPA, CENPB, CHAMP1, EPAS1,
Neuropsychiatric	18	ESR2, FOS, LHX2, LIN28B, PAX6, PPARD,
		PPARG, RBPJ, SKI, SOX5, SOX6, THAP1
		ARID3A, BCL6, CENPA, DDIT3, E2F1, FOXO1,
Respiratory	18	FOXO3, JUNB, MAX, MEOX2, NPAS1, PHF1,
		PITX1, RARA, RXRA, TFDP1, ZBTB16, ZNF467
Cardiovascular + Respiratory	2	ESR1, RELA
Cardiovascular + Neuropsychiatric	1	CREB5
Hematologic		BACH1, ELK3, ERG, ETS1, ETV3, GATAD2A,
+	16	GTF2B, GTF2I, HSF1, NCOA2, NFATC2,
Neuropsychiatric		RUNX1, SNAI1, THRA, ZBTB38, ZNF326
Neuropsychiatric + Respiratory	2	LTF, NR3C1
Hematologic + Respiratory	1	SMAD4
Hematologic +		
Respiratory +	5	HIF1A, MYC, STAT3, STAT5A, ZNF281
Neuropsychiatric		
Hematologic +		
Cardiovascular +	1	CREB1
Neuropsychiatric		
Hematologic + Respiratory +		
Cardiovascular +	1	ATF4
Neuropsychiatric +		

### The combined PPI network-graph coloring model highlights potential druggable host targets and therapies

**Fig. 6:**
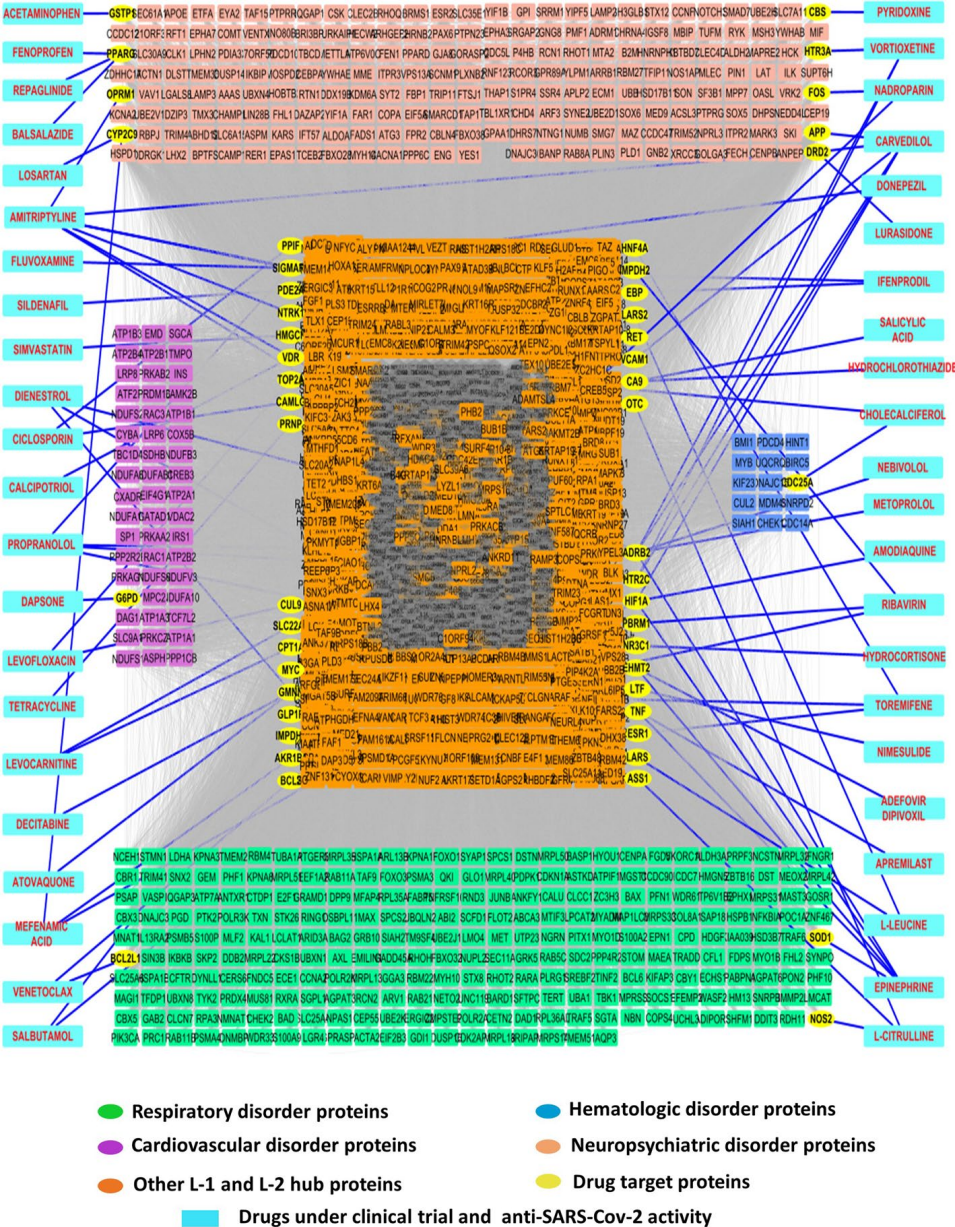
Druggable host protein recognition amongst L-1 and L-2 hub proteins. Viral drug target database mapping of hub proteins uncovers the highlighted (yellow color) druggable L-1 and L-2 hub proteins and their involvement in the pathophysiology of respiratory, cardiovascular, hematologic, and neuropsychiatric disorders

To evaluatethe therapeutic significance of the identified L-1 and L-2 hub proteins (Fig. 5A) for COVID-19 disease management, the druggability of hub proteins were screened for FDA-approved antiviral drug. A list of 59 such antiviral drugs were selected for screening based on the recent study in Zhou et al. [32]. A total of 44 drugs in this list were chosen based on their clinical trial evidence for SARS-CoV-2 and 23 for their anti-SARS-CoV-2 activities determined by at least two NCATS (National Center for Advancing Translational Sciences) defined assays. Eight drugs were commonly identified in both groups - used for clinical trial, and showed anti-SARS-CoV-2 activity in two NCATS-defined assays (SuppleAdditional file 2: Sheet S11). Druggable human targets of these 59 drugs were shortlisted from ChengF-Lab deposited repository (https://github.com/ChengF-Lab/AFnetproximity/blob/main/proximity, Accessed March, 2023). This list was mapped for druggability screening of L-1 and L-2 hub proteins. The result showed that among these 59 antiviral drugs, our combined PPI-graph coloring model identified 51 host interactors of 44 drugs as their therapeutic targets, which belong to L-1 and L-2 hub proteins (Fig. 6, yellow colored node), and is given in Table 4. It further validates the productivity of this combined PPI-graph coloring approach to predict potential therapeutic targets. Furthermore, the results highlighted 1, 1, 3 and 9 drug targets and respective 1, 1, 3, 13 drugs associated with cardiovascular, hematologic, respiratory, and neuropsychiatric disorders respectively. The remaining 37 drug targets for 26 drugs were identified as hub proteins but not associated with these four symptoms, which might be responsible for other COVID related functions. These pathobiology-associated drug targets and respective drugs might have added beneficial effects to resolve the COVID-19 associated pathobiology and the antiviral activity. According to the published study, these drugs could be categorized as anti-infective, anti-inflammatory, antihypertensive, and anti-neoplastic [32]. It suggests that these various types of drugs can resolve the disease through diverse mechanisms of action. Therefore, based on the assessment of candidate’s conditions, the expression pattern of the specific target host proteins, disease stage and associated pathobiology, the right drug might be predicted to treat them.

**Table 4:** Target proteins of drugs under clinical trials and with anti-SARS-CoV-2 activities

Drug category	Drug name	Direct host protein target
Clinical trial	Metoprolol	ADRB2
	Nebivolol	ADRB2
	Salicylic acid	CA9
	Pyridoxine	CBS
	Cholecalciferol	CDC25A, VDR
	Levocarnitine	CPT1A, SLC22A4
	Levofloxacin	CUL9, TOP2A
	Losartan	CYP2C9
	Ifenprodil	EBP, SIGMAR1
	Propranolol	EHMT2, SIGMAR1, HTR2C, ADRB2
	Dapsone	G6PD
	Salbutamol	GLP1R, ADRB2
	Atovaquone	GMNN
	Acetaminophen	GSTP1
	Simvastatin	HMGCR
	Vortioxetine	HTR3A, HTR2C, ADRB2
	L-Leucine	LARS, LARS2
	Nimesulide	LTF
	Nadroparin	MYC, FOS
	L-Citrulline	NOS2, OTC, ASS1
	Hydrocortisone	NR3C1
	Ribavirin	PBRM1, IMPDH2, IMPDH1
	Sildenafil	PDE2A
	Ciclosporin	PPIF, CAMLG
	Donepezil	SIGMAR1
	Fluvoxamine	SIGMAR1
	Epinephrine	SOD1, TNF, EHMT2, HIF1A, ADRB2
Common	Venetoclax	BCL2, BCL2L1
	Decitabine	GMNN
	Amodiaquine	HIF1A
	Mefenamic acid	HIF1A, CYP2C9, AKR1B1
	Toremifene	HNF4A, GMNN, ESR1
	Tetracycline	PRNP
	Apremilast	TNF
Anti SARS-CoV-2 activity	Dienestrol	ADRB2, GMNN, ESR1
	Amitriptyline	ADRB2, OPRM1, SIGMAR1, RET, NTRK1, DRD2, HTR2C
	Hydrochlorothiazide	CA9
	Lurasidone	DRD2
	Adefovir dipivoxil	EHMT2
	Carvedilol	HIF1A, VCAM1, DRD2, APP, HTR2C, ADRB2
	Balsalazide	PPARG
	Fenoprofen	PPARG
	Repaglinide	PPARG
	Calcipotriol	VDR

### Comparisons of our proposed model with other existing models

To further justify the applicability of graph coloring approach in computational network biology, we have compared the results of our proposed two level-PPI-Graph Coloring model with some recent studies (Tables 5, 6, 7). Some recent studies identified COVID-19-associated crucial hub proteins and related biological pathways, leading to various pathophysiology [7, 12, 31]. Likewise, our proposed model identifies hub proteins accountable for NIH-documented four types of disorders - cardiovascular, hematologic, neuropsychiatric, and respiratory. The authors highlighted that COVID-19-associated host genes and pathways are accountable for other viral infections [5, 8, 9]. Our proposed model also shows that, 80.4% of the L1 hub proteins interact with several virus proteins which belong to 9 different DNA and 18 different RNA viral family, other than SARS-CoV-2. On the other hand, 59% of the L2 hub proteins interact with other non-SARS-CoV-2 proteins belonging to 11 different DNA and 21 different RNA viral families. Existing research also predicted potential drug targets and respective drugs with anti-SARS-CoV-2 or anti-viral activities, some of them are under clinical trials [5, 12, 31, 32]. Likewise, our model also predicts 51 drug targets that belong to L1 or L2 hub proteins, along with 44 potential drugs with anti-SARS-CoV-2 activities, amongst which 34 are under clinical trials. It demonstrates the applicability of graph coloring in PPI network biology for identifying crucial hub proteins, affected biological pathways, drug targets and respective drugs in a comparable and competitive manner with other existing models.

**Table 5:** Comparisons of different methods in identifying SARS-CoV-2-associated hub proteins

Methods used	Number of identified hub prteins	
	Level-1	Level-2
Machine learning [5]	1326	Not applicable
Unsupervised machine learning [7]	1300	Not applicable
Receiver operating characteristic (ROC) curve analysis [8]	994	Not applicable
Network topological ananlysis [9]	15	Not applicable
Random walk with restart (RWR) [12]	476	Not applicable
Statistical R-packages (DESeq2 and edgeR) [31]	109	Not applicable
2-Level-PPI-graph coloring (this research work)	1082	1922

**Table 6:** Comparisons of different methods in identifying SARS-CoV-2-associated pathways

Methods used	Number of identified pathways	
	Level-1	Level-2
Machine learning [5]	7	Not applicable
Unsupervised machine learning [7]	95	Not applicable
Receiver operating characteristic (ROC) curve analysis [8]	165	Not applicable
Network topological ananlysis [9]	3	Not applicable
Random walk with restart (RWR) [12]	148	Not applicable
Statistical R-packages (DESeq2 and edgeR) [31]	42	Not applicable
2-Level-PPI-graph coloring (this research work)	38	112

**Table 7:** Comparison of different methods in drug discovery for therapeutic treatment of SARS-CoV-2

Method used	Number of identified drugs	Drugs under clinical trials not reported	Drugs with anti-SARS-CoV-2 activity not reported	Drugs with common features not reported	Number of host target 30
Machine learning [5]	12	Not reported	Not reported	Not reported	30
RWR [12]	130	40	30	Not reported	Not reported
DESeq2 and edgeR [31]	7	Not reported	Not reported	Not reported	15
Mass spectrometry [32]	59	44	23	8	Not reported
This research work	44	34	17	7	51

## Discussion

In this research, we established two levels of the PPI network and used graph coloring to pinpoint SARS-CoV-2-associated significant host interactors. Here, we first used direct human interactors of SARS-CoV-2 proteins from the BioGRID database to construct the first level of our PPI network. Later, we used the second level of human interactors of the first level’s hub proteins to create the second level of the PPI network. Finally, we implemented a graph coloring algorithm on each PPI network to distinguish L-1 and L-2 hub proteins from the first and second levels of the PPI network as the most promising facilitators of SARS-CoV-2 for developing the disease manifestations. This combined PPI-graph coloring approach sorted out 1,082 and 1,922 L-1 and L-2 hub proteins, respectively, which may have a significant relationship between SARS-CoV-2 infection, disease progression, and development of associated manifestations in the host body. To evaluate their significance, the HVIDB database search was carried out, and the results indicated that most of these hub proteins were equally significant in other RNA/DNA virus-human interactions characterized by previous studies. Among the reported interactions of L-1 hub proteins with other non-SARS-CoV-2 RNA viruses, the number of interactions with orthomyxoviridae (influenza family viruses), flaviviridae (Yellow Fever, Dengue Fever, Japanese encephalitis, West Nile viruses, and Zika virus) was the highest. Likewise, reported interactions of L-1 hub proteins with papilomaviridae and herpesviridae were the second highest among other non-SARS-CoV-2 DNA viruses. Similarly, reported interactions of L-2 hub proteins with other non-SARS-CoV-2 viruses revealed that the highest number of interactions with other RNA viruses were orthomyxoviriade, flaviviridae, and DNA viruses Herpesviridae, and retroviridae. The orthomyxoviridae and flaviviridae belong to a highly transmitted RNA virus family like SARS-CoV-2 and spread from other infected mammals through the air through coughs, sneezes, and arthropods vectors, respectively. Hence, the previous report of our approach specified L-1 and L-2 hub proteins in viral diseases signifying that those could also act as essential mediators of SARS-CoV-2 to develop the COVID-19-associated illness and symptoms. Therefore, our approach can filter out vital RNA-viral disease-associated hub proteins that could play a substantial role in SARS-CoV-2 infection and associated manifestations. It further indicated that the rest of the hub proteins, identified uniquely and had not been reported previously, could play a significant role only in SARS-CoV-2-associated disease manifestation.

After that, we analyzed cellular localization to evaluate the importance and association of each level of hub proteins (L-1 and L-2) with COVID-19. The result revealed that most of the L-1 hub proteins were cytosolic and plasma membrane residing, suggesting their higher probability of interaction with SARS-CoV-2 protein during infection and disease initiation. Whereas most of the L-2 hub proteins were nucleus or cytosolic residing proteins suggesting that those might be essential proteins through which SARS-CoV-2 alters the biological signals to develop pathological conditions. This hypothesis was further validated by examining the possibility of identifying transcription factors amongst them. Transcription factor database mapping confirmed that almost 11% of identified L-2 hub proteins were transcription factors, whereas none of the L-1 hub proteins were identified as TF. It suggests that the first level of host-interactors of SARS-CoV-2, possibly through these second levels of interactors, change the biological homeostasis and lead to the disease manifestations of COVID-19 in the host.

Furthermore, we analyzed pathway associations of L-1 and L-2 hub proteins to discover how SARS-CoV-2 altered the hosts’ biological pathways to establish the infection and develop the disease symptoms. KEGG pathway analysis highlighted MAPK, cAMP, cGMP-PKG, FOXO, mTOR, AMPK, HIF-1, ErbB, and p53 signaling among the enriched 55 involved pathways connected with L-1 and L-2 hub proteins. MAPK signaling pathway is an essential regulator of various cellular processes, including cell proliferation, differentiation, apoptosis, metabolism, stress response, and inflammation (https://www.kegg.jp/pathway/map=map04010 &keyword=MAPK, Accessed April, 2023), [47]. Therefore, alteration of the MAPK pathway could be the possible way SARS-CoV-2 can exploit the host cellular signaling for their proliferation and bypass the host immune defense to establish the infection. Multiple studies have reported the involvement of the MAPK kinase pathway in COVID-19 and the development of associated symptoms [6, 48]. cAMP signaling pathway regulates cell growth and differentiation, gene transcription, protein expression, metabolism, neurotransmitter synthesis, growth factors, and muscle contraction (https://www.genome.jp/pathway/ko04024, Accessed April, 2023), [49, 50]. cAMP pathway acts as a master regulator of immune cell functions, including inflammation, phagocytosis, and killing intracellular pathogens [51, 52]. Hence alteration of this pathway could be a possible target of SARS-CoV-2 to bypass the innate immune defense in the lung and can develop cardiac dysfunction by altering muscle functionality, catecholamine downstream signaling, and response to cardiac hypoxia [53]. cGMP-PKG pathway regulates many cellular processes, including relaxation and contraction of vascular smooth muscle cells, cardiac hypertrophy, and vascular injury/restenosis (https://www.genome.jp/pathway/hsa04022, Accessed April, 2023), [54]. Modulation of the NO-cGMP-PDE5 pathway is reported in COVID-19 patients for developing pulmonary fibrosis and inflammation in the lung and cardiovascular disorders [54, 55]. FOXO signaling pathway is another important pathway that can regulate multiple essential biological processes, including cell-cycle regulation, apoptosis, glucose metabolism, oxidative stress resistance, DNA repair, immune regulation, and muscle atrophy (https://www.genome.jp/pathway/hsa04068, Accessed April, 2023), [47]. Earlier studies stated that SARS-CoV-2 exploits FOXO signaling pathway to alter host inflammatory and immunological response [56]. The mTOR signaling pathway is essential to respond against environmental cues to boost the metabolism and maintain the bioenergetics based on nutrient availability and modulate protein synthesis. Accordingly, it can regulate cell growth, proliferation and angiogenesis, autophagy, and apoptosis (https://www.genome.jp/pathway/map04150, Accessed April, 2023), [57]. Impaired mTOR signaling also became apparent in COVID-19 patients through which SARS-CoV-2 regulates their life cycle and modulates inflammation [58, 59]. mTOR pathway-inhibiting drugs also demonstrated their efficacy as potential therapeutic agents for COVID-19 [60]. The HIF-1 signaling pathway is the master regulator of oxygen homeostasis and consequently regulates the metabolism. The HIF-1 pathway significantly maintains lung and heart functionality (https://www.genome.jp/pathway/hsa04066, Accessed April, 2023), [61]. Therefore, impairment of this pathway commonly leads to pulmonary and cardiovascular disorders that are identified in COVID-19 patients. So, targeting this pathway using novel targeted therapies is considered as an improved management strategy for lung injury and cardiac disorders of COVID-19 patients [62]. The ErbB pathway also plays a vital role in various cellular functions, including cell division, adhesion, migration, differentiation, and death (https://www.genome.jp/pathway/hsa04012, Accessed April, 2023), [63]. Likewise, AMPK signaling pathway coordinates cell growth, metabolism, and autophagy, and the p53 pathway regulates cellular homeostasis, DNA replication, chromosome segregation, and cell division [64, 65]. Thus, L-1 and L-2 hub proteins detected by our PPI-graph coloring approach could be the plausible mediators through which SARS-CoV-2 can change the diverse host biological functionality to establish the infection, disease progression, and development of disease symptoms.

To further specify the involvement of L-1 and L-2 hub proteins in developing COVID-19 disease symptoms, we categorized them based on their functional association with respiratory, cardiovascular, hematologic, and neuropsychiatric disorders. These four symptoms were the most established manifestations of COVID-19. Our results showed that these four symptoms were associated with good numbers of L-1 and L-2 hub proteins. We found that 18, 5, 1, and 18 transcription factors were accompanied by respiratory, cardiovascular, hematologic, and neuropsychiatric disorders identified as L-2 hub proteins. It suggests that those transcription factors can play an essential role in regulating their target genes to develop the symptoms in response to SARS-CoV-2 infection and other associated L-1 and L-2 hub proteins. An additional literature search about the functional implications of transcription factors for developing the related symptoms showed their significance. Likewise, we searched the practical importance of respiratory disorders-related transcription factors (ARID3A, BCL6, E2F1, FOXO1, FOXO3, RARA, RXRA, CENPA, DDIT3, JUNB, MAX, MEOX2, NPAS1, PHF1, PITX1, TFDP1, ZBTB16, ZNF467) identified by or network to validate their importance in COVID-19 respiratory disorders development. ARID3A (A-T rich interacting domain 3a) can bind the DNA of its target gene and modulate the gene regulations. It is reported that ARID3A plays an essential role in antibody production, and type-1 IFN production of antigen-activated B cells also helps maintain B cell homeostasis [66, 67]. Type-1 IFNs and B-cell antibody secretion are essential for the immune response against interstellar pathogens like viruses. So, altering this transcription factor can be beneficial for SARS-CoV-2 as an escape mechanism from immune clearance. It is also reported that in COVID-19 asymptomatic patients, ARID3A is strongly associated with IFN or IFN receptors suggesting the importance of this TF [68]. Similarly, BCL6 is a master regulator of T follicular helper cells proliferation, germinal center formation, development, and function of B cells. It also regulates the function of neutrophils and macrophages and is essential in developing a humoral response against virus infection. So the alteration of these genes can modulate the humoral response in favor of viral survivability as reported in COVID-19 or influenza-infected patients [69, 70]. E2F1 is a critical transcription factor that regulates cell cycle progression, DNA-damage response [71], apoptosis [72], senescence [73], and metabolism [74]. So the alteration of this transcription factor can alter the cellular fate of lung cells to develop respiratory disorders, which are evident in SARS-CoV-2 infection as well [75, 76]. Moreover, it is also apparent that SARS-CoV-2 can use E2F1 as one of the host transcription factors for expressing the viral genes, which are essential for their infectivity [77]. Likewise, forkhead transcription factors of the O class family transcription factors including FOXO1, FOXO3 regulate an array of cellular functions like proliferation, differentiation, survival, metabolism, oxidative stress, apoptosis, secretion of inflammatory cytokines, development and maturation of B and T lymphocytes, and more [78]. It is also crucial in maintaining lung physiology and regulating the immune and inflammatory response against infection. So dysregulation of these transcription factors can develop respiratory disorders [79, 80]. Retinol depletion and retinoid signaling disorders have been identified as essential mechanisms in COVID-19 pathogenesis characterized by defects in Type-I interferon synthesis and consequent immune system dysregulation and severe inflammation development. Hence the alteration of retinoic acid receptors (RARs), including RARA and related Retinoid X Receptors (RXRs) including RXRA can facilitate COVID-19 pathogenesis which is evident in the published reports [81, 82]. Accordingly, it is reported that SARS-CoV-2 infection exploits the FOXO regulatory pathway to develop the disease and associated lung and respiratory disorders [56, 83]. Similarly, we found the relevant evidence of alteration or association of transcription factors CENPA [84], DDIT3 [85], JUNB [86, 87], TFDP1 [88], ZBTB16 [89], ZNF467 [90] for the alteration of diverse cellar process and function to develop the COVID-19 pathogenesis and associated respiratory disorders. Literature search information implies that the transcription factors identified by our approach have significance in COVID-19 pathogenesis and developing related symptoms. This further validates the usefulness of using our PPI-graph coloring approach for detecting essential proteins for other viral diseases.

The remarkable research for successful COVID-19 vaccine development has resulted in safe and potential vaccines to overcome the pandemic. However, developing potential drugs to treat patients with severe disease symptoms during and after COVID is still significant for patients’ health. Accordingly, we investigated the druggability of L-1 and L-2 hub proteins for 59 drugs which either used for SARS-CoV-2 clinical trials or had anti-SARS-CoV-2 activity [32]. We found 51 druggable targets, identified as either L-1 or L-2 hub proteins of 44 drugs. Some of them were identified as disease-associated symptoms category of respiratory, cardiovascular, hematologic, and neuropsychiatric disorders. We did a literature search about those drugs and their target genes to evaluate further the importance of those druggable targets and drugs and their most probable significance in COVID-19. Respiratory symptoms associated with druggable genes were NOS2, SOD1, and BCL2L1; probable drugs were L-Citrulline, Epinephrine, and Venetoclax, respectively. L-citrulline is a non-essential amino acid that can be converted to L-arginine and then to nitric oxide (NO) through nitric oxide synthases (NOSs). Nitric oxide acts as an essential biological free radical in maintaining vascular tone, immune functions, inflammation, and oxidative stress. NO deficiency in COVID-19 patients correlates with disease severity, and a supplement of L-citrulline can maintain the citrulline/arginine/NO balance through NOS and could be the beneficial treatment option for severely symptomatic patients [91, 92]. Venetoclax is a BH3-mimetic that inhibits BCL-2protein function and induces apoptosis [93]. Alteration of redox homeostasis through the alteration of sodium dismutase (SOD1), an enzyme that catalyzes the conversion of superoxide to hydrogen peroxide and oxygen, is reported in COVID-19 patients [94, 95]. Alteration of redox equilibrium of immune cells can lead to severe inflammation and cytokine storm, respiratory disorder, and cardiovascular collapse. So targeting SOD1 through epinephrine treatment might benefit those patients [96, 97]. BCL2L1 is an anti-apoptotic protein that regulates cell death and is highly upregulated in inflammatory tissue. The published reports also show BCL2L1 upregulates in COVID-19 patients [98, 99]. Similarly, the other druggable genes and respective drugs might be good options to treat patients with severe disease symptoms.

## Conclusions

In this work, applicability of evolutionary graph coloring in computational network biology has been demonstrated. We first developed an algorithm using differential evolution for solving graph coloring problem, and tested the performance of the algorithm on some widely-used standard benchmark graph instances. Upon obtaining promising results within acceptable time, the same approach has been incorporated in network biology and a generic model has been proposed to highlight the plausible missing links between viral infection and the development of associated human pathophysiology. We tested the model on SARS-CoV-2 infection to identify the possible mediators responsible of this viral infection and associated disease manifestations in human bodies. Accordingly, we established two levels of protein–protein interaction networks. We applied graph coloring algorithm and degree centrality criteria on both the PPI networks and identified the crucial first and second levels of hub proteins as vital mediators of COVID-19 manifestations. To confirm their biological significance in COVID-19 pathophysiology, we performed corresponding DAVID functional analysis, interactions with other RNA and DNA viral families, KEGG pathway enrichment analysis, transcription factors identification, and their association with COVID-19 symptoms. Furthermore, we analyzed their druggable target identifications and predicted the respective drugs to corroborate their therapeutical importance by cross-validating the druggability of the hub proteins against the existing literature and FDA-approved antiviral drugs. To the best of our knowledge, this is the first novel 2-level PPI-graph coloring approach to illustrate the more profound insight of SARS-CoV-2 infection and associated human pathophysiology and identification of probable drugs and respective drug targets.

Although this research helps to narrow down the crucial mediators of SARS-CoV-2 through which it establishes the infection, develops the inflammation and other disease symptoms, and predicts plausible targets and respective drugs, a further in-depth biological experimental study using proper in vitro and in vivo models is required to validate those findings. Also, the performance of the proposed model can also be validated for some existing known infectious diseases. Furthermore, it can also be applied to analyze the more profound insight of disease manifestations and drug identification of new viral infections.

### Supplementary Information

**Additional file 1. **NIH-reported literatures list.

**Additional file 2. **Table: Sheet S1–S11.

## Data Availability

The proposed process workflow is created with Professional Science Figure Creation Software (https://www.biorender.com/, Accessed April, 2023). During the current study, the datasets are generated from the publicly avaiable Biogrid repository (https://thebiogrid.org/search.php?search=SARS-CoV-2* &organism=2697049, Accessed March, 2022). The results are are analyzed using DAVID functional annotation tool (https://david.ncifcrf.gov/tools.jsp, Accessed January, 2023). All data generated or analysed during this study are included in this article and its supplementary information files.

## Footnotes

1 https://covid19.who.int/, Last Access: 4 November, 2023.
