# Supplementary material for: Generic model to unravel the deeper insights of viral infections: an empirical application of evolutionary graph coloring in computational network biology
Source: BMC Bioinformatics. 2024 Feb 16;25:74. doi: 10.1186/s12859-024-05690-0 (PMC10874019; doi:10.1186/s12859-024-05690-0)
Supplement: Supplementary file 1 — Additional file 1. NIH-reported literatures list. [file 12859_2024_5690_MOESM1_ESM.pdf]

# Lung Fibrosis:

Y. Mitsuuchi, J. R. Testa, Cytogenetics and molecular genetics of lung cancer, American Journal of Medical Genetics 115 (3) (2002) 183–188.

Z. Shaozhang, L. Xiaomei, Z. Aiping, H. Jianbo, S. Xiangqun, Y. Qi- tao, Detection of EML4-ALK fusion genes in non-small cell lung cancer patients with clinical features associated with EGFR mutations, Genes, Chromosomes and Cancer 51 (10) (2012) 925–932.

U. A. Sartorius, P. H. Krammer, Upregulation of bcl-2 is involved in the mediation of chemotherapy resistance in human small cell lung cancer cell lines, International Journal of Cancer 97 (5) (2002) 584–592.

N. Shivapurkar, J. Reddy, P. M. Chaudhary, A. F. Gazdar, Apoptosis and lung cancer: A review, Journal of Cellular Biochemistry 88 (5) (2003) 885–898.

J. Ekedahl, B. Joseph, M. Y. Grigoriev, M. Müller, C. Magnusson, R. Lewensohn, B. Zhivotovsky, Expression of inhibitor of apoptosis pro- teins in small- and non-small-cell lung carcinoma cells, Experimental Cell Research 279 (2) (2002) 277–290.

G. P. Pfeifer, R. Dammann, Methylation of the tumor suppressor gene RASSF1a in human tumors, Biochemistry (Moscow) 70 (5) (2005) 576–583.

S. Toyooka, T. Mitsudomi, J. Soh, K. Aokage, M. Yamane, T. Oto, K. Kiura, S. Miyoshi, Molecular oncology of lung cancer, General Thoracic and Cardiovascular Surgery 59 (8) (2011) 527–537.

Y. Wang, P. J. Kuan, C. Xing, J. T. Cronkhite, F. Torres, R. L. Rosen- blatt, J. M. DiMaio, L. N. Kinch, N. V. Grishin, C. K. Garcia, Genetic defects in surfactant protein a2 are associated with pulmonary fibrosis and lung cancer, The American Journal of Human Genetics 84 (1) (2009) 52–59.

A. PANANI, C. ROUSSOS, Cytogenetic and molecular aspects of lung cancer, Cancer Letters 239 (1) (2006) 1–9.

T. Reungwetwattana, S. J. Weroha, J. R. Molina, Oncogenic path- ways, molecularly targeted therapies, and highlighted clinical trials in non–small-cell lung cancer (NSCLC), Clinical Lung Cancer 13 (4) (2012) 252–266.

R. Ferrara, N. Auger, E. Auclin, B. Besse, Clinical and translational im- plications of RET rearrangements in non–small cell lung cancer, Journal of Thoracic Oncology 13 (1) (2018) 27–45.

S. Popat, D. Gonzalez, T. Min, J. Swansbury, M. Dainton, J. G. Croud, A. J. Rice, A. G. Nicholson, ALK translocation is associated with ALK immunoreactivity and extensive signet-ring morphology in primary lung adenocarcinoma, Lung Cancer 75 (3) (2012) 300–305.

R. Roskoski, Anaplastic lymphoma kinase (ALK): Structure, oncogenic activation, and pharmacological inhibition, Pharmacological Research 68 (1) (2013) 68–94.

F. J. Kaye, Molecular biology of lung cancer, Lung Cancer 34 (2001) S35– S41.

M. Sanchez-Cespedes, Dissecting the genetic alterations involved in lung carcinogenesis, Lung Cancer 40 (2) (2003) 111–121.

F. R. Hirsch, G. V. Scagliotti, C. J. Langer, M. Varella-Garcia, W. A. Franklin, Epidermal growth factor family of receptors in preneoplasia and lung cancer: perspectives for targeted therapies, Lung Cancer 41 (2003) 29–42.

C. Spruck, H. Strohmaier, M. Watson, A. P. Smith, A. Ryan, W. Krek, S. I. Reed, A CDK-independent function of mammalian cks1, Molecular Cell 7 (3) (2001) 639–650.

R. C. Buttery, R. C. Rintoul, T. Sethi, Small cell lung cancer: the importance of the extracellular matrix, The International Journal of Biochemistry & Cell Biology 36 (7) (2004) 1154–1160.

R. C. Rintoul, T. Sethi, The role of extracellular matrix in small-cell lung cancer, The Lancet Oncology 2 (7) (2001) 437–442.

J. Yokota, M. Nishioka, M. Tani, T. Kohno, Genetic alterations responsible for metastatic phenotypes of lung cancer cells, Clinical & experimental metastasis 20 (3) (2003) 189.

L. Altucci, H. Gronemeyer, The promise of retinoids to fight against cancer, Nature Reviews Cancer 1 (3) (2001) 181–193.

M. Soda, Y. L. Choi, M. Enomoto, S. Takada, Y. Yamashita, S. Ishikawa, S. ichiro Fujiwara, H. Watanabe, K. Kurashina, H. Hatanaka, M. Bando, S. Ohno, Y. Ishikawa, H. Aburatani, T. Niki, Y. Sohara, Y. Sugiyama, H. Mano, Identification of the transforming EML4–ALK fusion gene in non-small-cell lung cancer, Nature 448 (7153) (2007) 561–566.

P. A. Jänne, N. Gray, J. Settleman, Factors underlying sensitivity of cancers to small-molecule kinase inhibitors, Nature Reviews Drug Discovery 8 (9) (2009) 709–723.

S. Adhikary, M. Eilers, Transcriptional regulation and transformation by myc proteins, Nature Reviews Molecular Cell Biology 6 (8) (2005) 635–645.

H. Osada, T. Takahashi, Genetic alterations of multiple tumor suppressors and oncogenes in the carcinogenesis and progression of lung cancer, Oncogene 21 (48) (2002) 7421–7434.

Y. Zhang, I. Noth, J. G. Garcia, N. Kaminski, A variant in the pro- moter of muc5b and idiopathic pulmonary fibrosis, New England Journal of Medicine 364 (16) (2011) 1576–1577.

A. K. Virmani, A. Rathi, S. Zochbauer-Muller, N. Sacchi, Y. Fukuyama,D. Bryant, A. Maitra, S. Heda, K. M. Fong, F. Thunnissen, J. D. Minna, A. F. Gazdar, Promoter methylation and silencing of the retinoic acid receptor-gene in lung carcinomas, JNCI Journal of the National Cancer Institute 92 (16) (2000) 1303–1307.

W. E. Lawson, J. E. Loyd, A. L. Degryse, Genetics in pulmonary fibrosis familial cases provide clues to the pathogenesis of idiopathic pul- monary fibrosis, The American Journal of the Medical Sciences 341 (6) (2011) 439–443.

A. Yoshida, K. Tsuta, H. Nakamura, T. Kohno, F. Takahashi, H. Asamura, I. Sekine, M. Fukayama, T. Shibata, K. Furuta, H. Tsuda, Comprehensive histologic analysis of ALK-rearranged lung carcinomas, American Journal of Surgical Pathology 35 (8) (2011) 1226–1234.

H. Awaya, Gene amplification and protein expression of EGFR and HER2 by chromogenic in situ hybridisation and immunohistochemistry in atypi- cal adenomatous hyperplasia and adenocarcinoma of the lung, Journal of Clinical Pathology 58 (10) (2005) 1076–1080.

Y. L. Choi, K. Takeuchi, M. Soda, K. Inamura, Y. Togashi, S. Hatano, M. Enomoto, T. Hamada, H. Haruta, H. Watanabe, et al., Identification of novel isoforms of the eml4-alk transforming gene in non–small cell lung cancer, Cancer research 68 (13) (2008) 4971–4976.

R. Breuer, P. Postmus, E. Smit, Molecular pathology of non-small-cell lung cancer, Respiration 72 (3) (2005) 313–330.

W. D. Travis, U. Costabel, D. M. Hansell, T. E. King, D. A. Lynch, A. G. Nicholson, C. J. Ryerson, J. H. Ryu, M. Selman, A. U. Wells, J. Behr,D. Bouros, K. K. Brown, T. V. Colby, H. R. Collard, C. R. Cordeiro, V. Cottin, B. Crestani, M. Drent, R. F. Dudden, J. Egan, K. Flaherty, C. Hogaboam, Y. Inoue, T. Johkoh, D. S. Kim, M. Kitaichi, J. Loyd, F. J. Martinez, J. Myers, S. Protzko, G. Raghu, L. Richeldi, N. Sverzellati, J. Swigris, D. Valeyre, An official american thoracic society/european res- piratory society statement: Update of the international multidisciplinary classification of the idiopathic interstitial pneumonias, American Journal of Respiratory and Critical Care Medicine 188 (6) (2013) 733–748.

S. T. Lehtonen, A. Veijola, H. Karvonen, E. Lappi-Blanco, R. Sormunen, S. Korpela, U. Zagai, M. C. Sköld, R. Kaarteenaho, Pirfenidone and nintedanib modulate properties of fibroblasts and myofibroblasts in id- iopathic pulmonary fibrosis, Respiratory Research 17 (1).

S. Heinrich, D. Hartl, M. Griese, Surfactant protein a - from genes to human lung diseases, Current Medicinal Chemistry 13 (27) (2006) 3239–3252.

K. Takeuchi, Discovery stories of RET fusions in lung cancer: A mini- review, Frontiers in Physiology 10.

M. Santoro, M. Moccia, G. Federico, F. Carlomagno, RET gene fusions in malignancies of the thyroid and other tissues, Genes 11 (4) (2020) 424.

S. Aviel-Ronen, F. H. Blackhall, F. A. Shepherd, M.-S. Tsao, K-ras mutations in non-small-cell lung carcinoma: A review, Clinical Lung Cancer 8 (1) (2006) 30–38.

V. Koudelakova, M. Kneblova, R. Trojanec, J. Drabek, M. Hajduch, Non- small cell lung cancer - genetic predictors, Biomedical Papers 157 (2) (2013) 125–136.

M. C. Emblom-Callahan, M. K. Chhina, O. A. Shlobin, S. Ahmad, E. S. Reese, E. P. Iyer, D. N. Cox, R. Brenner, N. A. Burton, G. M. Grant, et al., Genomic phenotype of non-cultured pulmonary fibroblasts in idiopathic pulmonary fibrosis, Genomics 96 (3) (2010) 134–145.

Y. Li, L. Niu, Identification of the effects of covid-19 on patients with pulmonary fibrosis and lung cancer: a bioinformatics analysis and literature review, Scientific Reports 12 (1) (2022) 1–17.

J. A. Kropski, T. S. Blackwell, J. E. Loyd, The genetic basis of idiopathic pulmonary fibrosis, European Respiratory Journal 45 (6) (2015) 1717–1727.

C. Gu, X. Shi, X. Dang, J. Chen, C. Chen, Y. Chen, X. Pan, T. Huang, Identification of common genes and pathways in eight fibrosis diseases, Frontiers in Genetics 11 (2021) 627396.

A. Kaur, S. K. Mathai, D. A. Schwartz, Genetics in idiopathic pulmonary fibrosis pathogenesis, prognosis, and treatment, Frontiers in medicine 4 (2017) 154.

# Post Bacterial Pneumonia:

X. Dai, L. Zhang, T. Hong, Host cellular signaling induced by influenza virus, Science China Life Sciences 54 (1) (2011) 68–74.

T. T. Thanh, H. R. van Doorn, M. D. de Jong, Human h5n1 influenza: Current insight into pathogenesis, The International Journal of Biochem- istry & Cell Biology 40 (12) (2008) 2671–2674.

C. Ehrhardt, R. Seyer, E. R. Hrincius, T. Eierhoff, T. Wolff, S. Ludwig, Interplay between influenza a virus and the innate immune signaling, Mi- crobes and Infection 12 (1) (2010) 81–87.

B. Ramakrishnan, K. Viswanathan, K. Tharakaraman, V. Danˇc´ık, R. Ra- man, G. J. Babcock, Z. Shriver, R. Sasisekharan, A structural and math- ematical modeling analysis of the likelihood of antibody-dependent en- hancement in influenza, Trends in Microbiology 24 (12) (2016) 933–943.

G. K. Atkin-Smith, M. Duan, W. Chen, I. K. H. Poon, The induction and consequences of influenza a virus-induced cell death, Cell Death & Disease 9 (10)

J. S. Long, B. Mistry, S. M. Haslam, W. S. Barclay, Host and viral determinants of influenza a virus species specificity, Nature Reviews Microbiology 17 (2) (2018) 67–81.

X. Lu, A. Masic, Y. Li, Y. Shin, Q. Liu, Y. Zhou, The PI3k/akt pathway inhibits influenza a virus-induced bax-mediated apoptosis by negatively regulating the JNK pathway via ASK1, Journal of General Virology 91 (6) (2010) 1439–1449.

T. O. Edinger, M. O. Pohl, S. Stertz, Entry of influenza a virus: host factors and antiviral targets, Journal of General Virology 95 (2) (2014) 263–277.

K. Sharma, S. Tripathi, P. Ranjan, P. Kumar, R. Garten, V. Deyde, J. M. Katz, N. J. Cox, R. B. Lal, S. Sambhara, et al., Influenza a virus nucle- oprotein exploits hsp40 to inhibit pkr activation, PloS one 6 (6) (2011) e20215.

D. Paterson, E. Fodor, Emerging roles for the influenza a virus nuclear export protein (NEP), PLoS Pathogens 8 (12) (2012) e1003019.

T. Ichinohe, A. Iwasaki, H. Hasegawa, Innate sensors of influenza virus: clues to developing better intranasal vaccines, Expert Review of Vaccines 7 (9) (2008) 1435–1445.

B. G. Hale, R. A. Albrecht, A. Garc´ıa-Sastre, Innate immune evasion strategies of influenza viruses, Future Microbiology 5 (1) (2010) 23–41.

Y. Fan, S. Sanyal, R. Bruzzone, Breaking bad: How viruses subvert the cell cycle, Frontiers in Cellular and Infection Microbiology 8.

X. Chen, S. Liu, M. U. Goraya, M. Maarouf, S. Huang, J.-L. Chen, Host immune response to influenza a virus infection, Frontiers in Immunology 9.

D. Dou, R. Revol, H. Ö stbye, H. Wang, R. Daniels, Influenza a virus cell entry, replication, virion assembly and movement, Frontiers in Immunology 9.

A. Nogales, L. Martinez-Sobrido, D. Topham, M. DeDiego, Modulation of innate immune responses by the influenza a NS1 and PA-x proteins, Viruses 10 (12) (2018) 708.

C. E. van de Sandt, J. H. C. M. Kreijtz, G. F. Rimmelzwaan, Evasion of influenza a viruses from innate and adaptive immune responses, Viruses 4 (9) (2012) 1438–1476.

J. Li, M. Yu, W. Zheng, W. Liu, Nucleocytoplasmic shuttling of influenza a virus proteins, Viruses 7 (5) (2015) 2668–2682.

V. L. Sage, A. Cinti, R. Amorim, A. Mouland, Adapting the stress response: Viral subversion of the mTOR signaling pathway, Viruses 8 (6) (2016) 152.

P. Michael, D. Brabant, F. Bleiblo, C. V. Ramana, M. Rutherford, S. Khu- rana, T. Tai, A. Kumar, A. Kumar, Influenza a induced cellular signal transduction pathways, Journal of thoracic disease 5 (Suppl 2) (2013) S132.

Z. T. Varga, P. Palese, The influenza a virus protein PB1-f2, Virulence 2 (6) (2011) 542–546.

# Pulmonary Embolism:

Y. Mitsuuchi, J. R. Testa, Cytogenetics and molecular genetics of lung cancer, American Journal of Medical Genetics 115 (3) (2002) 183–188.

Z. Shaozhang, L. Xiaomei, Z. Aiping, H. Jianbo, S. Xiangqun, Y. Qi- tao, Detection of EML4-ALK fusion genes in non-small cell lung cancer patients with clinical features associated with EGFR mutations, Genes, Chromosomes and Cancer 51 (10) (2012) 925–932.

G. P. Pfeifer, R. Dammann, Methylation of the tumor suppressor gene RASSF1a in human tumors, Biochemistry (Moscow) 70 (5) (2005) 576–583.

S. Toyooka, T. Mitsudomi, J. Soh, K. Aokage, M. Yamane, T. Oto, K. Kiura, S. Miyoshi, Molecular oncology of lung cancer, General Thoracic and Cardiovascular Surgery 59 (8) (2011) 527–537.

A. PANANI, C. ROUSSOS, Cytogenetic and molecular aspects of lung cancer, Cancer Letters 239 (1) (2006) 1–9.

T. Reungwetwattana, S. J. Weroha, J. R. Molina, Oncogenic pathways, molecularly targeted therapies, and highlighted clinical trials in non–small-cell lung cancer (NSCLC), Clinical Lung Cancer 13 (4) (2012) 252–266.

R. Ferrara, N. Auger, E. Auclin, B. Besse, Clinical and translational im- plications of RET rearrangements in non–small cell lung cancer, Journal of Thoracic Oncology 13 (1) (2018) 27–45.

S. Popat, D. Gonzalez, T. Min, J. Swansbury, M. Dainton, J. G. Croud, A. J. Rice, A. G. Nicholson, ALK translocation is associated with ALK immunoreactivity and extensive signet-ring morphology in primary lung adenocarcinoma, Lung Cancer 75 (3) (2012) 300–305.

R. Roskoski, Anaplastic lymphoma kinase (ALK): Structure, oncogenic activation, and pharmacological inhibition, Pharmacological Research 68 (1) (2013) 68–94.

F. R. Hirsch, G. V. Scagliotti, C. J. Langer, M. Varella-Garcia, W. A. Franklin, Epidermal growth factor family of receptors in preneoplasia and lung cancer: perspectives for targeted therapies, Lung Cancer 41 (2003) 29–42.

J. Yokota, M. Nishioka, M. Tani, T. Kohno, Genetic alterations responsible for metastatic phenotypes of lung cancer cells, Clinical & experimental metastasis 20 (3) (2003) 189.

L. Altucci, H. Gronemeyer, The promise of retinoids to fight against cancer, Nature Reviews Cancer 1 (3) (2001) 181-193.

M. Soda, Y. L. Choi, M. Enomoto, S. Takada, Y. Yamashita, S. Ishikawa, S. ichiro Fujiwara, H. Watanabe, K. Kurashina, H. Hatanaka, M. Bando, S. Ohno, Y. Ishikawa, H. Aburatani, T. Niki, Y. Sohara, Y. Sugiyama, H. Mano, Identification of the transforming EML4–ALK fusion gene in non-small-cell lung cancer, Nature 448 (7153) (2007) 561–566.

P. A. Jänne, N. Gray, J. Settleman, Factors underlying sensitivity of can- cers to small-molecule kinase inhibitors, Nature Reviews Drug Discovery 8 (9) (2009) 709–723.

H. Osada, T. Takahashi, Genetic alterations of multiple tumor suppres- sors and oncogenes in the carcinogenesis and progression of lung cancer, Oncogene 21 (48) (2002) 7421–7434.

A. K. Virmani, A. Rathi, S. Zochbauer-Muller, N. Sacchi, Y. Fukuyama, D. Bryant, A. Maitra, S. Heda, K. M. Fong, F. Thunnissen, J. D. Minna, A. F. Gazdar, Promoter methylation and silencing of the retinoic acid receptor- gene in lung carcinomas, JNCI Journal of the National Cancer Institute 92 (16) (2000) 1303–1307.

A. Yoshida, K. Tsuta, H. Nakamura, T. Kohno, F. Takahashi, H. Asamura, I. Sekine, M. Fukayama, T. Shibata, K. Furuta, H. Tsuda, Comprehensive histologic analysis of ALK-rearranged lung carcinomas, American Journal of Surgical Pathology 35 (8) (2011) 1226–1234.

H. Awaya, Gene amplification and protein expression of EGFR and HER2 by chromogenic in situ hybridisation and immunohistochemistry in atypical adenomatous hyperplasia and adenocarcinoma of the lung, Journal of Clinical Pathology 58 (10) (2005) 1076–1080.

Y. L. Choi, K. Takeuchi, M. Soda, K. Inamura, Y. Togashi, S. Hatano, M. Enomoto, T. Hamada, H. Haruta, H. Watanabe, et al., Identification of novel isoforms of the eml4-alk transforming gene in non–small cell lung cancer, Cancer research 68 (13) (2008) 4971–4976.

R. Breuer, P. Postmus, E. Smit, Molecular pathology of non-small-cell lung cancer, Respiration 72 (3) (2005) 313–330.

K. Takeuchi, Discovery stories of RET fusions in lung cancer: A mini- review, Frontiers in Physiology 10.

M. Santoro, M. Moccia, G. Federico, F. Carlomagno, RET gene fusions in malignancies of the thyroid and other tissues, Genes 11 (4) (2020) 424.

S. Aviel-Ronen, F. H. Blackhall, F. A. Shepherd, M.-S. Tsao, K-ras mu- tations in non-small-cell lung carcinoma: A review, Clinical Lung Cancer 8 (1) (2006) 30–38.

V. Koudelakova, M. Kneblova, R. Trojanec, J. Drabek, M. Hajduch, Non-small cell lung cancer - genetic predictors, Biomedical Papers 157 (2) (2013) 125–136.

Y. Peng, T. Wang, Y. Zheng, A. Lian, D. Zhang, Z. Xiong, Z. Hu, K. Xia, C. Shu, A novel variation of SERPINC1 caused deep venous thrombosis in a chinese family, Medicine 98 (1) (2019) e13999.

M. D. Do, D. V. Pham, L. P. Le, L. H. Gia Le, L. B. Minh Tran, M. D. Dang Huynh, Q. M. Do, H. A. Vu, N. H. Nguyen, T. P. Mai, Recurrent proc and novel pros1 mutations in vietnamese patients diagnosed with idiopathic deep venous thrombosis, International journal of laboratory hematology 43 (2) (2021) 266–272.

F. ROSENDAAL, P. REITSMA, Genetics of venous thrombosis, Journal of Thrombosis and Haemostasis 7 (2009) 301–304.

D. Scarvelis, P. S. Wells, Diagnosis and treatment of deep-vein thrombosis, Canadian Medical Association Journal 175 (9) (2006) 1087–1092.

S. J. Halliday, D. T. Matthews, M. H. Talati, E. D. Austin, Y. R. Su, T. S. Absi, N. L. Fortune, D. Gailani, A. Matafonov, J. D. West, et al., A multifaceted investigation into molecular associations of chronic thromboembolic pulmonary hypertension pathogenesis, JRSM Cardiovascular Disease 9 (2020) 2048004020906994.

# Anxiety:

X. Xu, B. Gao, X. Li, S. Lei, Study on the antianxiety mechanism of suanzaoren decoction based on network pharmacology and molecular docking, Evidence-Based Complementary and Alternative Medicine 2021 (2021) 1–28.

O. I. Rudko, A. V. Tretiakov, E. A. Naumova, E. A. Klimov, et al., Role of ppars in progression of anxiety: literature analysis and signaling pathways reconstruction, PPAR research 2020.

X. Su, W. Li, L. Lv, X. Li, J. Yang, X.-J. Luo, J. Liu, Transcriptome- wide association study provides insights into the genetic component of gene expression in anxiety, Frontiers in Genetics 12 (2021) 740134.

K. L. Purves, J. R. Coleman, S. M. Meier, C. Rayner, K. A. Davis, R. Cheesman, M. Baekvad-Hansen, A. D. Børglum, S. Wan Cho, J. Jürgen Deckert, et al., A major role for common genetic variation in anxiety disorders, Molecular psychiatry 25 (12) (2020) 3292–3303.

M. G. Gottschalk, K. Domschke, Genetics of generalized anxiety disorder and related traits, Dialogues in clinical neuroscience.

# Cerebral Vein Thrombosis:

L. Gromadziński, Ł. Paukszto, A. Skowrońska, P. Holak, M. Smoliński, E. Ł opieńska-Biernat, E. Lepiarczyk, A. Lipka, J. P. Jastrzḱebski, M. Ma- jewska, Transcritomic profiling of femoral veins in deep vein thrombosis in a porcine model, Cells 10 (7) (2021) 1576.

P. Liu, W. Jiang, H. Zhang, Identification of target gene of venous thromboembolism in patients with lymphoma via microarray analysis, Oncology Letters 14 (3) (2017) 3313–3318.

P. Qi, M. Huang, T. Li, Screening the potential biomarkers of covid-19- related thrombosis through bioinformatics analysis, Frontiers in genetics (2022) 1335.

# Depression:

H. Daniel, Cellular mechanisms of cerebellar LTD, Trends in Neuro- sciences 21 (9) (1998) 401–407.

C. Levenes, H. Daniel, F. Crépel, Long-term depression of synaptic trans- mission in the cerebellum: cellular and molecular mechanisms revisited, Progress in Neurobiology 55 (1) (1998) 79–91.

F. Metzger, J. P. Kapfhammer, Protein kinase c: its role in activity- dependent purkinje cell dendritic development and plasticity, The Cere- bellum 2 (3) (2003) 206–214.

M. Ito, Cerebellar long-term depression: Characterization, signal trans- duction, and functional roles, Physiological Reviews 81 (3) (2001) 1143–1195.

C.-F. Kao, P. Jia, Z. Zhao, P.-H. Kuo, Enriched pathways for major de- pressive disorder identified from a genome-wide association study, The The International Journal of Neuropsychopharmacology 15 (10) (2012) 1401–1411.

D. Qi, K. Chen, Bioinformatics analysis of potential biomarkers and pathway identification for major depressive disorder, Computational and Mathematical Methods in Medicine 2021 (2021) 1–11.

G. Zhang, S. Xu, Z. Zhang, Y. Zhang, Y. Wu, J. An, J. Lin, Z. Yuan, L. Shen, T. Si, Identification of key genes and the pathophysiology associated with major depressive disorder patients based on integrated bioinformatics analysis, Frontiers in psychiatry 11 (2020) 192.

B. Zhao, Q. Fan, J. Liu, A. Yin, P. Wang, W. Zhang, Identification of key modules and genes associated with major depressive disorder in adolescents, Genes 13 (3) (2022) 464.

R. Geng, X. Huang, Identification of major depressive disorder disease- related genes and functional pathways based on system dynamic changes of network connectivity, BMC medical genomics 14 (2021) 1–11.

S. Sall, W. Thompson, A. Santos, D. S. Dwyer, Analysis of major depression risk genes reveals evolutionary conservation, shared phenotypes, and extensive genetic interactions, Frontiers in psychiatry 12 (2021) 698029.

N. Mariani, N. Cattane, C. Pariante, A. Cattaneo, Gene expression stud- ies in depression development and treatment: an overview of the underlying molecular mechanisms and biological processes to identify biomarkers, Translational psychiatry 11 (1) (2021) 354.

# Insomnia:


B. Enanga, R. Burchmore, M. Stewart, M. Barrett, Sleeping sickness and the brain, Cellular and Molecular Life Sciences 59 (5) (2002) 845–858.

E. Pays, B. Vanhollebeke, Human innate immunity against african trypanosomes, Current Opinion in Immunology 21 (5) (2009) 493–498.

S. Bisser, O. Ouwe-Missi-Oukem-Boyer, F. Toure, Z. Taoufiq, B. Bouteille, A. Buguet, D. Mazier, Harbouring in the brain: A focus on immune eva- sion mechanisms and their deleterious effects in malaria and human african trypanosomiasis, International Journal for Parasitology 36 (5) (2006) 529–540.

W. Masocha, M. E. Rottenberg, K. Kristensson, Migration of african trypanosomes across the blood–brain barrier, Physiology & Behavior 92 (1-2) (2007) 110–114.

K. Kristensson, M. Nygård, G. Bertini, M. Bentivoglio, African trypanosome infections of the nervous system: Parasite entry and effects on sleep and synaptic functions, Progress in Neurobiology 91 (2) (2010) 152–171.

J. Donelson, Multiple mechanisms of immune evasion by african trypanosomes, Molecular and Biochemical Parasitology 91 (1) (1998) 51–66.

J. D. Lonsdale-Eccles, D. J. Grab, Trypanosome hydrolases and the blood–brain barrier, Trends in Parasitology 18 (1) (2002) 17–19.

D. J. Grab, P. G. Kennedy, Traversal of human and animal trypanosomes across the blood-brain barrier, Journal of Neurovirology 14 (5) (2008) 344–351.

B. Vanhollebeke, E. Pays, The trypanolytic factor of human serum: many ways to enter the parasite, a single way to kill, Molecular Microbiology 76 (4) (2010) 806–814.

N. Antoine-Moussiaux, P. Buscher, D. Desmecht, Host-parasite interac- tions in trypanosomiasis: on the way to an antidisease strategy, Infection and Immunity 77 (4) (2009) 1276–1284.

D. J. Grab, J. C. Garcia-Garcia, O. V. Nikolskaia, Y. V. Kim, A. Brown, C. A. Pardo, Y. Zhang, K. G. Becker, B. A. Wilson, A. P. C. de A. Lima, J. Scharfstein, J. S. Dumler, Protease activated receptor signaling is re- quired for african trypanosome traversal of human brain microvascular endothelial cells, PLoS Neglected Tropical Diseases 3 (7) (2009) e479.

T. Liu, G. Wang, X. Zhang, X. Liu, Z. Liang, X. Ren, D. Yan, W. Zhang, B serum proteome profiles revealed dysregulated proteins and mechanisms associated with insomnia patients: A preliminary study, Frontiers in Integrative Neuroscience 16.

H.-J. Ban, S. C. Kim, J. Seo, H.-B. Kang, J. K. Choi, Genetic and metabolic characterization of insomnia, PloS one 6 (4) (2011) e18455.

M. J. Lind, P. R. Gehrman, Genetic pathways to insomnia, Brain sciences 6 (4) (2016) 64.

P. R. Gehrman, C. Pfeiffenberger, E. M. Byrne, The role of genes in the insomnia phenotype, Sleep medicine clinics 8 (3) (2013) 323–331.

# Post Traumatic Stress Disorder:

P.-F. Kuan, X. Yang, X. Ren, C. Che, M. Waszczuk, R. Kotov, S. Clous- ton, P. K. Singh, S. T. Glenn, E. C. Gomez, et al., Mapping the transcrip- tomics landscape of post-traumatic stress disorder symptom dimensions in world trade center responders, Translational Psychiatry 11 (1) (2021) 310.

S. M. Hemmings, P. Swart, J. S. Womersely, E. S. Ovenden, L. L. van den Heuvel, N. W. McGregor, S. Meier, S. Bardien, S. Abrahams, G. Tromp, et al., Rna-seq analysis of gene expression profiles in posttraumatic stress disorder, parkinson's disease and schizophrenia identifies roles for common and distinct biological pathways, Discover Mental Health 2 (1) (2022) 6.

Y. Bian, L. Yang, M. Zhao, Z. Li, Y. Xu, G. Zhou, W. Li, L. Zeng, Identification of key genes and pathways in post-traumatic stress disorder using microarray analysis, Frontiers in psychology 10 (2019) 302.

B. Anna, D. Avetyan, L. Hovhannisyan, G. Mkrtchyan, Gentics of post- traumatic stress disorder—candidate genes and their implication in the disease-associated molecular pathomechanisms, A Fresh Look at Anxiety Disorders; IntechOpen: London, UK (2015) 65–88.

M. E. Garrett, X. J. Qin, D. Mehta, M. F. Dennis, C. E. Marx, G. A. Grant, V. M.-A. M. Workgroup, P. Initiative, Injury, T. S. I. C. Con- sortium, P. G. C. P. Group, et al., Gene expression analysis in three posttraumatic stress disorder cohorts implicates inflammation and innate immunity pathways and uncovers shared genetic risk with major depres- sive disorder, Frontiers in neuroscience 15 (2021) 678548.

M. C. Cornelis, N. R. Nugent, A. B. Amstadter, K. C. Koenen, Genetics of post-traumatic stress disorder: review and recommendations for genome- wide association studies, Current psychiatry reports 12 (2010) 313–326.

H. F. Guill´en-Burgos, K. Guti´errez-Ruiz, Genetic advances in post- traumatic stress disorder, Revista Colombiana de Psiquiatr´ıa (English ed.) 47 (2) (2018) 108–118.

# Seizures:


S. E. Heron, H. A. Phillips, J. C. Mulley, A. Mazarib, M. Y. Neufeld, S. F. Berkovic, I. E. Scheffer, Genetic variation of CACNA1h in idiopathic generalized epilepsy, Annals of Neurology 55 (4) (2004) 595–596.

A. Kapoor, P. Satishchandra, R. Ratnapriya, R. Reddy, J. Kadandale, S. K. Shankar, A. Anand, An idiopathic epilepsy syndrome linked to 3q13.3-q21 and missense mutations in the extracellular calcium sensing receptor gene, Annals of Neurology 64 (2) (2008) 158–167.

T. Arsov, S. A. Mullen, S. Rogers, A. M. Phillips, K. M. Lawrence, J. A. Damiano, H. Goldberg-Stern, Z. Afawi, S. Kivity, C. Trager, S. Petrou, S. F. Berkovic, I. E. Scheffer, Glucose transporter 1 deficiency in the idiopathic generalized epilepsies, Annals of Neurology 72 (5) (2012) 807–815.

A. Salzmann, M. Guipponi, P. J. Lyons, L. D. Fricker, M. Sapio, C. Lam- bercy, C. Buresi, B. O. A. Bencheikh, F. Lahjouji, R. Ouazzani, A. Cre- spel, D. Chaigne, A. Malafosse, Carboxypeptidase a6 gene (CPA6) mu- tations in a recessive familial form of febrile seizures and temporal lobe epilepsy and in sporadic temporal lobe epilepsy, Human Mutation 33 (1) (2011) 124–135.

J. Nakayama, Progress in searching for the febrile seizure susceptibility genes, Brain and Development 31 (5) (2009) 359–365.

G. Rudolf, G. Lesca, M. M. Mehrjouy, A. Labalme, M. Salmi, I. Bache, N. Bruneau, M. Pendziwiat, J. Fluss, J. de Bellescize, J. Scholly, R. S. Møller, D. Craiu, N. Tommerup, M. P. Valenti-Hirsch, C. Schluth-Bolard, F. Sloan-B´ena, K. L. Helbig, S. Weckhuysen, P. Edery, S. Coulbaut, M. Abbas, I. E. Scheffer, S. Tang, C. T. Myers, H. Stamberger, G. L. Carvill, D. N. Shinde, H. C. Mefford, E. Neagu, R. Huether, H.-M. Lu, A. Dica, J. S. Cohen, C. Iliescu, C. Pomeran, J. Rubenstein, I. Helbig, D. Sanlaville, E. Hirsch, P. Szepetowski, Loss of function of the retinoid- related nuclear receptor (RORB) gene and epilepsy, European Journal of Human Genetics 24 (12) (2016) 1761–1770.

K. Haug, M. Warnstedt, A. K. Alekov, T. Sander, A. Ram´ırez, B. Poser, S. Maljevic, S. Hebeisen, C. Kubisch, J. Rebstock, S. Horvath, K. Hall- mann, J. S. Dullinger, B. Rau, F. Haverkamp, S. Beyenburg, H. Schulz, D. Janz, B. Giese, G. Müller-Newen, P. Propping, C. E. Elger, C. Fahlke, H. Lerche, A. Heils, Mutations in CLCN2 encoding a voltage-gated chlo- ride channel are associated with idiopathic generalized epilepsies, Nature Genetics 33 (4) (2003) 527–532.

X. Li, S. Poschmann, Q. Chen, W. Fazeli, N. J. Oundjian, F. M. Snoeijen- Schouwenaars, O. Fricke, E.-J. Kamsteeg, M. Willemsen, Q. K. Wang, De novo BK channel variant causes epilepsy by affecting voltage gating but not ca2+ sensitivity, European Journal of Human Genetics 26 (2) (2018) 220–229.

M. Mantegazza, A. Gambardella, R. Rusconi, E. Schiavon, F. Annesi, R. R. Cassulini, A. Labate, S. Carrideo, R. Chifari, M. P. Canevini, et al., Identification of an nav1. 1 sodium channel (scn1a) loss-of-function mu- tation associated with familial simple febrile seizures, Proceedings of the National Academy of Sciences 102 (50) (2005) 18177–18182.

A. Escayg, M. D. Waard, D. D. Lee, D. Bichet, P. Wolf, T. Mayer, J. John- ston, R. Baloh, T. Sander, M. H. Meisler, Coding and noncoding variation of the human calcium-channel $\beta$4-subunit gene CACNB4 in patients with idiopathic generalized epilepsy and episodic ataxia, The American Journal of Human Genetics 66 (5) (2000) 1531–1539.

L. M. Dibbens, GABRD encoding a protein for extra- or peri-synaptic GABAA receptors is a susceptibility locus for generalized epilepsies, Hu- man Molecular Genetics 13 (13) (2004) 1315–1319.

C. Marini, I. E. Scheffer, K. M. Crossland, B. E. Grinton, F. L. Phillips, J. M. McMahon, S. J. Turner, J. T. Dean, S. Kivity, A. Mazarib, M. Y. Neufeld, A. D. Korczyn, L. A. Harkin, L. M. Dibbens, R. H. Wallace, J. C. Mulley, S. F. Berkovic, Genetic architecture of idiopathic generalized epilepsy: Clinical genetic analysis of 55 multiplex families, Epilepsia 45 (5) (2004) 467–478.

P. Lachance-Touchette, P. Brown, C. Meloche, P. Kinirons, L. Lapointe, H. Lacasse, A. Lortie, L. Carmant, F. Bedford, D. Bowie, P. Cossette, Novel $\alpha 1$ and $\gamma 2$ GABAA receptor subunit mutations in families with idiopathic generalized epilepsy, European Journal of Neuroscience 34 (2) (2011) 237–249.

Y. G. Weber, H. Lerche, Genetic mechanisms in idiopathic epilepsies, Developmental Medicine & Child Neurology 50 (9) (2008) 648–654.

M. Boillot, M. Morin-Brureau, F. Picard, S. Weckhuysen, V. Lambrecq, C. Minetti, P. Striano, F. Zara, M. Iacomino, S. Ishida, et al., Novel gabrg2 mutations cause familial febrile seizures, Neurology Genetics 1 (4).

R. M. Piro, I. Molineris, U. Ala, F. Di Cunto, Evaluation of candidate genes from orphan feb and gefs+ loci by analysis of human brain gene expression atlases, PLoS One 6 (8) (2011) e23149.

Y. Nakamura, X. Shi, T. Numata, Y. Mori, R. Inoue, C. Lossin, T. Z. Baram, S. Hirose, Novel HCN2 mutation contributes to febrile seizures by shifting the channel s kinetics in a temperature-dependent manner, PLoS ONE 8 (12) (2013) e80376.

K. T. Kahle, N. D. Merner, P. Friedel, L. Silayeva, B. Liang, A. Khanna, Y. Shang, P. Lachance-Touchette, C. Bourassa, A. Levert, et al., Genet- ically encoded impairment of neuronal kcc 2 cotransporter function in human idiopathic generalized epilepsy, EMBO reports 15 (7) (2014) 766–774.

J. Y. Han, H. J. Lee, Y.-M. Lee, J. Park, Identification of missense AD- GRV1 mutation as a candidate genetic cause of familial febrile seizure 4, Children 7 (9) (2020) 144.

J. Wang, Z.-J. Lin, L. Liu, H.-Q. Xu, Y.-W. Shi, Y.-H. Yi, N. He, W.-P. Liao, Epilepsy-associated genes, Seizure 44 (2017) 11–20.

W. Guo, D.-M. Shang, J.-H. Cao, K. Feng, Y.-C. He, Y. Jiang, S. Wang, Y.-F. Gao, Identifying and analyzing novel epilepsy-related genes using random walk with restart algorithm, BioMed research international 2017.

O. K. Steinlein, Genetics and epilepsy, Dialogues in clinical neuroscience.

A. Fernández-Marmiesse, I. Roca, F. D´ıaz-Flores, V. Cantar´ın, M. S. P´erez-Poyato, A. Fontalba, F. Laranjeira, S. Quintans, O. Moldovan, B. Felgueroso, et al., Rare variants in 48 genes account for 42% of cases of epilepsy with or without neurodevelopmental delay in 246 pediatric patients, Frontiers in neuroscience 13 (2019) 1135.

# Stroke:

L. Pu, M. Wang, K. Li, T. Feng, P. Zheng, S. Li, Y. Yao, L. Jin, Identification micro-rnas functional modules and genes of ischemic stroke based on weighted gene co-expression network analysis (wgcna), Genomics 112 (4) (2020) 2748–2754.

X. Diao, A. Liu, Identification of core pathways based on attractor and crosstalk in ischemic stroke, Experimental and Therapeutic Medicine 15 (2) (2018) 1520–1524.

Q. Xie, X. Zhang, S. Peng, J. Sun, X. Chen, Y. Deng, L. Yi, Identification of novel biomarkers in ischemic stroke: a genome-wide integrated analysis, BMC medical genetics 21 (2020) 1–13.

E. Terni, N. Giannini, M. Brondi, V. Montano, U. Bonuccelli, M. Man- cuso, Genetics of ischaemic stroke in young adults, BBA clinical 3 (2015) 96–106.

M. Dichgans, S. L. Pulit, J. Rosand, Stroke genetics: discovery, biology, and clinical applications, The Lancet Neurology 18 (6) (2019) 587–599.

A. Lindgren, Stroke genetics: a review and update, Journal of stroke 16 (3) (2014) 114.

A. Ekkert, A. Šliachtenko, J. Grigaite˙, B. Burnyte˙, A. Utkus, D. Jatuˇzis, Ischemic stroke genetics: what is new and how to apply it in clinical practice?, Genes 13 (1) (2021) 48.

H. S. Markus, Stroke genetics, Human molecular genetics 20 (R2) (2011) R124–R131.

J.-m. Guo, A.-j. Liu, D.-f. Su, Genetics of stroke, Acta Pharmacologica Sinica 31 (9) (2010) 1055–1064.

# Arrhythmias:


G. Wallukat, Die $\beta$-adrenergen rezeptoren, Herz 27 (2002) 683–690.

T. Herren, P. A. Gerber, F. Duru, Arrhythmogenic right ventricular car- diomyopathy/dysplasia: a not so rare "disease of the desmosome" with multiple clinical presentations, Clinical Research in Cardiology 98 (3) (2009) 141–158.

D. Ho, L. Yan, K. Iwatsubo, D. E. Vatner, S. F. Vatner, Modulation of $\beta$-adrenergic receptor signaling in heart failure and longevity: targeting adenylyl cyclase type 5, Heart failure reviews 15 (2010) 495–512.

S. P. Barry, S. M. Davidson, P. A. Townsend, Molecular regulation of cardiac hypertrophy, The International Journal of Biochemistry & Cell Biology 40 (10) (2008) 2023–2039.

A. R. Soltis, J. J. Saucerman, Synergy between camkii substrates and $\beta$-adrenergic signaling in regulation of cardiac myocyte ca2+ handling, Biophysical journal 99 (7) (2010) 2038–2047.

J. M. Lapp´e, C. M. Pelfrey, W. W. Tang, Recent insights into the role of autoimmunity in idiopathic dilated cardiomyopathy, Journal of Cardiac Failure 14 (6) (2008) 521–530.

H. Yamashita, Myosin light chain isoforms modify force-generating ability of cardiac myosin by changing the kinetics of actin–myosin interaction, Cardiovascular Research 60 (3) (2003) 580–588.

A. Phrommintikul, N. Chattipakorn, Roles of cardiac ryanodine recep- tor in heart failure and sudden cardiac death, International Journal of Cardiology 112 (2) (2006) 142–152.

F. Triposkiadis, G. Karayannis, G. Giamouzis, J. Skoularigis, G. Louridas, J. Butler, The sympathetic nervous system in heart failure, Journal of the American College of Cardiology 54 (19) (2009) 1747–1762.

J. J. Saucerman, A. D. McCulloch, Mechanistic systems models of cell sig- naling networks: a case study of myocyte adrenergic regulation, Progress in Biophysics and Molecular Biology 85 (2-3) (2004) 261–278.

M. Kuzumoto, A. Takeuchi, H. Nakai, C. Oka, A. Noma, S. Matsuoka, Simulation analysis of intracellular na+ and cl- homeostasis during $\beta$1- adrenergic stimulation of cardiac myocyte, Progress in biophysics and molecular biology 96 (1-3) (2008) 171–186.

M. S. Parmacek, R. Solaro, Biology of the troponin complex in cardiac myocytes, Progress in Cardiovascular Diseases 47 (3) (2004) 159–176.

M. Zheng, W. Zhu, Q. Han, R.-P. Xiao, Emerging concepts and therapeu- tic implications of $\beta$-adrenergic receptor subtype signaling, Pharmacology & therapeutics 108 (3) (2005) 257–268.

M. C. Schaub, M. A. Hefti, M. Zaugg, Integration of calcium with the signaling network in cardiac myocytes, Journal of Molecular and Cellular Cardiology 41 (2) (2006) 183–214.

M. Grimm, J. H. Brown, $\beta$-adrenergic receptor signaling in the heart: Role of CaMKII, Journal of Molecular and Cellular Cardiology 48 (2) (2010) 322–330.

J. Mason, Myocarditis and dilated cardiomyopathy an inflammatory link, Cardiovascular Research 60 (1) (2003) 5–10.

W.-M. Franz, O. J. Müller, H. A. Katus, Cardiomyopathies: from genetics to the prospect of treatment, The Lancet 358 (9293) (2001) 1627–1637.

D. M. Bers, Cardiac excitation–contraction coupling, Nature 415 (6868) (2002) 198–205.

J. A. Towbin, N. E. Bowles, The failing heart, Nature 415 (6868) (2002) 227–233.

M. M. Awad, H. Calkins, D. P. Judge, Mechanisms of disease: molecular genetics of arrhythmogenic right ventricular dysplasia/cardiomyopathy, Nature Clinical Practice Cardiovascular Medicine 5 (5) (2008) 258–267.

X. H. T. Wehrens, A. R. Marks, Novel therapeutic approaches for heart failure by normalizing calcium cycling, Nature Reviews Drug Discovery 3 (7) (2004) 565–574.

P. E. Tam, Coxsackievirus myocarditis: Interplay between virus and host in the pathogenesis of heart disease, Viral Immunology 19 (2) (2006) 133–146.

J. S. Berg, B. C. Powell, R. E. Cheney, A millennial myosin census, Molecular Biology of the Cell 12 (4) (2001) 780–794.

J. P. van Tintelen, R. M. Hofstra, A. C. Wiesfeld, M. P. van den Berg, R. N. Hauer, J. D. Jongbloed, Molecular genetics of arrhythmogenic right ventricular cardiomyopathy: emerging horizon?, Current Opinion in Cardiology 22 (3) (2007) 185–192.

S. SEN-CHOWDHRY, P. SYRRIS, W. J. McKENNA, Genetics of right ventricular cardiomyopathy, Journal of Cardiovascular Electrophysiology 16 (8) (2005) 927–935.

J. LINGREL, A. MOSELEY, I. DOSTANIC, M. COUGNON, S. HE, P. JAMES, A. WOO, K. O CONNOR, J. NEUMANN, Functional roles of the $\alpha$ isoforms of the na, k-ATPase, Annals of the New York Academy of Sciences 986 (1) (2003) 354–359.

T. J. Pritchard, E. G. Kranias, Junctin and the histidine-rich ca2+ binding protein: potential roles in heart failure and arrhythmogenesis, The Journal of physiology 587 (13) (2009) 3125–3133.

R. Xiao, $\beta$-adrenergic signaling in the heart: Dual coupling of the $\beta$2- adrenergic receptor to gs and gi, Proteins. Science 2001.

A. Luk, E. Ahn, G. S. Soor, J. Butany, Dilated cardiomyopathy: a review, Journal of Clinical Pathology 62 (3) (2008) 219–225.

F. Ahmad, J. Seidman, C. E. Seidman, THE GENETIC BASIS FOR CARDIAC REMODELING, Annual Review of Genomics and Human Ge- netics 6 (1) (2005) 185–216.

D. M. Bers, Calcium cycling and signaling in cardiac myocytes, Annual Review of Physiology 70 (1) (2008) 23–49.

D. Fatkin, R. M. Graham, Molecular mechanisms of inherited cardiomyopathies, Physiological Reviews 82 (4) (2002) 945–980.

A. M. Gordon, E. Homsher, M. Regnier, Regulation of contraction in striated muscle, Physiological Reviews 80 (2) (2000) 853–924.

R.-P. Xiao, H. Cheng, Y.-Y. Zhou, M. Kuschel, E. G. Lakatta, Recent advances in cardiac $\beta$2-adrenergic signal transduction, Circulation research 85 (11) (1999) 1092–1100.

J. H. Yang, J. J. Saucerman, Computational models reduce complexity and accelerate insight into cardiac signaling networks, Circulation Re- search 108 (1) (2011) 85–97.

A. P. Landstrom, D. Dobrev, X. H. Wehrens, Calcium signaling and cardiac arrhythmias, Circulation Research 120 (12) (2017) 1969–1993.

P. P. Liu, J. W. Mason, Advances in the understanding of myocarditis, Circulation 104 (9) (2001) 1076–1082.

R. Jahns, V. Boivin, L. Hein, S. Triebel, C. E. Angermann, G. Ertl, M. J. Lohse, Direct evidence for a $\beta$1-adrenergic receptor–directed autoimmune attack as a cause of idiopathic dilated cardiomyopathy, Journal of Clinical Investigation 113 (10) (2004) 1419–1429.

M. Yano, Y. Ikeda, M. Matsuzaki, Altered intracellular ca2+ handling in heart failure, Journal of Clinical Investigation 115 (3) (2005) 556–564.

C. A. MacRae, Arrhythmogenic right ventricular cardiomyopathy: moving toward mechanism, Journal of Clinical Investigation 116 (7) (2006) 1825–1828.

M. Endoh, Cardiac ca2+ signaling and ca2+ sensitizers, Circulation Journal 72 (12) (2008) 1915–1925.

T. Yoshikawa, A. Baba, Y. Nagatomo, Autoimmune mechanisms under- lying dilated cardiomyopathy, Circulation Journal 73 (4) (2009) 602–607.

D. M. Bers, S. Despa, Cardiac myocytes ca2+ and na+ regulation in normal and failing hearts, Journal of Pharmacological Sciences 100 (5) (2006) 315–322.

M. Endoh, Signal transduction and ca2+ signaling in intact myocardium, Journal of Pharmacological Sciences 100 (5) (2006) 525–537.

P. Zhao, Pathogenesis and therapy of autoimmunity-induced dilated cardiomyopathy, Frontiers in Bioscience Volume (14) (2009) 1708.

P. Taggart, M. R. Boyett, S. Logantha, P. D. Lambiase, Anger, emotion, and arrhythmias: From brain to heart, Frontiers in Physiology 2.

B. Gray, E. R. Behr, New insights into the genetic basis of inherited arrhythmia syndromes, Circulation: Cardiovascular Genetics 9 (6) (2016) 569–577.

A. J. Marian, B. Asatryan, X. H. Wehrens, Genetic basis and molecular biology of cardiac arrhythmias in cardiomyopathies, Cardiovascular research 116 (9) (2020) 1600–1619.

M. Wang, X. Tu, The genetics and epigenetics of ventricular arrhythmias in patients without structural heart disease, Frontiers in Cardiovascular Medicine 9.

M. T. Keating, M. C. Sanguinetti, Molecular and cellular mechanisms of cardiac arrhythmias, Cell 104 (4) (2001) 569–580.

# Congestive Heart Failure:

G. Wallukat, Die $\beta$-adrenergen rezeptoren, Herz 27 (2002) 683–690.

D. Ho, L. Yan, K. Iwatsubo, D. E. Vatner, S. F. Vatner, Modulation of $\beta$-adrenergic receptor signaling in heart failure and longevity: targeting adenylyl cyclase type 5, Heart failure reviews 15 (2010) 495–512.

S. P. Barry, S. M. Davidson, P. A. Townsend, Molecular regulation of cardiac hypertrophy, The International Journal of Biochemistry & Cell Biology 40 (10) (2008) 2023–2039.

A. R. Soltis, J. J. Saucerman, Synergy between camkii substrates and $\beta$-adrenergic signaling in regulation of cardiac myocyte ca2+ handling, Biophysical journal 99 (7) (2010) 2038–2047.

J. M. Lapp´e, C. M. Pelfrey, W. W. Tang, Recent insights into the role of autoimmunity in idiopathic dilated cardiomyopathy, Journal of Cardiac Failure 14 (6) (2008) 521–530.

F. Triposkiadis, G. Karayannis, G. Giamouzis, J. Skoularigis, G. Louridas, J. Butler, The sympathetic nervous system in heart failure, Journal of the American College of Cardiology 54 (19) (2009) 1747–1762.

J. J. Saucerman, A. D. McCulloch, Mechanistic systems models of cell signaling networks: a case study of myocyte adrenergic regulation, Progress in Biophysics and Molecular Biology 85 (2-3) (2004) 261–278.

M. Kuzumoto, A. Takeuchi, H. Nakai, C. Oka, A. Noma, S. Matsuoka, Simulation analysis of intracellular na+ and cl- homeostasis during $\beta$1- adrenergic stimulation of cardiac myocyte, Progress in biophysics and molecular biology 96 (1-3) (2008) 171–186.

M. Zheng, W. Zhu, Q. Han, R.-P. Xiao, Emerging concepts and therapeutic implications of $\beta$-adrenergic receptor subtype signaling, Pharmacology & therapeutics 108 (3) (2005) 257–268.

M. C. Schaub, M. A. Hefti, M. Zaugg, Integration of calcium with the signaling network in cardiac myocytes, Journal of Molecular and Cellular Cardiology 41 (2) (2006) 183–214.

M. Grimm, J. H. Brown, $\beta$-adrenergic receptor signaling in the heart: Role of CaMKII, Journal of Molecular and Cellular Cardiology 48 (2) (2010) 322–330.

J. Mason, Myocarditis and dilated cardiomyopathy an inflammatory link, Cardiovascular Research 60 (1) (2003) 5–10.

W.-M. Franz, O. J. Müller, H. A. Katus, Cardiomyopathies: from genetics to the prospect of treatment, The Lancet 358 (9293) (2001) 1627–1637.

D. M. Bers, Cardiac excitation–contraction coupling, Nature 415 (6868) (2002) 198–205.

J. A. Towbin, N. E. Bowles, The failing heart, Nature 415 (6868) (2002) 227–233.

X. H. T. Wehrens, A. R. Marks, Novel therapeutic approaches for heart failure by normalizing calcium cycling, Nature Reviews Drug Discovery 3 (7) (2004) 565–574.

P. E. Tam, Coxsackievirus myocarditis: Interplay between virus and host in the pathogenesis of heart disease, Viral Immunology 19 (2) (2006) 133–146.

R. Xiao, $\beta$-adrenergic signaling in the heart: Dual coupling of the $\beta$2- adrenergic receptor to gs and gi, Proteins. Science 2001.

A. Luk, E. Ahn, G. S. Soor, J. Butany, Dilated cardiomyopathy: a review, Journal of Clinical Pathology 62 (3) (2008) 219–225.

D. M. Bers, Calcium cycling and signaling in cardiac myocytes, Annual Review of Physiology 70 (1) (2008) 23–49.

D. Fatkin, R. M. Graham, Molecular mechanisms of inherited cardiomyopathies, Physiological Reviews 82 (4) (2002) 945–980.

R.-P. Xiao, H. Cheng, Y.-Y. Zhou, M. Kuschel, E. G. Lakatta, Recent advances in cardiac $\beta$2-adrenergic signal transduction, Circulation research 85 (11) (1999) 1092–1100.

J. H. Yang, J. J. Saucerman, Computational models reduce complexity and accelerate insight into cardiac signaling networks, Circulation Re- search 108 (1) (2011) 85–97.

P. P. Liu, J. W. Mason, Advances in the understanding of myocarditis, Circulation 104 (9) (2001) 1076–1082.

R. Jahns, V. Boivin, L. Hein, S. Triebel, C. E. Angermann, G. Ertl, M. J. Lohse, Direct evidence for a $\beta$1-adrenergic receptor–directed autoimmune attack as a cause of idiopathic dilated cardiomyopathy, Journal of Clinical Investigation 113 (10) (2004) 1419–1429.

M. Yano, Y. Ikeda, M. Matsuzaki, Altered intracellular ca2+ handling in heart failure, Journal of Clinical Investigation 115 (3) (2005) 556–564.

T. Yoshikawa, A. Baba, Y. Nagatomo, Autoimmune mechanisms under- lying dilated cardiomyopathy, Circulation Journal 73 (4) (2009) 602–607.

P. Zhao, Pathogenesis and therapy of autoimmunity-induced dilated cardiomyopathy, Frontiers in Bioscience Volume (14) (2009) 1708.

P. Taggart, M. R. Boyett, S. Logantha, P. D. Lambiase, Anger, emotion, and arrhythmias: From brain to heart, Frontiers in Physiology 2.

M. Ganapathi, L. Argyriou, F. Mart´ınez-Azor´ın, S. Morlot, G. Yigit, T. M. Lee, B. Auber, A. von Gise, D. S. Petrey, H. Thiele, et al., Bi-allelic mis- sense disease-causing variants in rpl3l associate neonatal dilated cardiomyopathy with muscle-specific ribosome biogenesis, Human genetics 139 (11) (2020) 1443–1454.

A.-K. Arndt, S. Schafer, J.-D. Drenckhahn, M. K. Sabeh, E. R. Plovie, A. Caliebe, E. Klopocki, G. Musso, A. A. Werdich, H. Kalwa, M. Heinig, R. F. Padera, K. Wassilew, J. Bluhm, C. Harnack, J. Martitz, P. J. Barton, M. Greutmann, F. Berger, N. Hubner, R. Siebert, H.-H. Kramer, S. A. Cook, C. A. MacRae, S. Klaassen, Fine mapping of the 1p36 deletion syndrome identifies mutation of PRDM16 as a cause of cardiomyopathy, The American Journal of Human Genetics 93 (1) (2013) 67–77.

M. G. Posch, M. J. Posch, C. Geier, B. Erdmann, W. Mueller, A. Richter, V. Ruppert, S. Pankuweit, B. Maisch, A. Perrot, J. Buttgereit, R. Di- etz, W. Haverkamp, C. Ö zcelik, A missense variant in desmoglein-2 pre- disposes to dilated cardiomyopathy, Molecular Genetics and Metabolism 95 (1-2) (2008) 74–80.

A. Levitas, E. Muhammad, G. Harel, A. Saada, V. C. Caspi, E. Manor, J. C. Beck, V. Sheffield, R. Parvari, Familial neonatal isolated cardiomy- opathy caused by a mutation in the flavoprotein subunit of succinate de-hydrogenase, European Journal of Human Genetics 18 (10) (2010) 1160–1165.

T. Meyer, , V. Ruppert, S. Ackermann, A. Richter, A. Perrot, S. R. Sperling, M. G. Posch, B. Maisch, S. Pankuweit, Novel mutations in the sarcomeric protein myopalladin in patients with dilated cardiomyopathy, European Journal of Human Genetics 21 (3) (2012) 294–300.

E. G. Jones, N. Mazaheri, R. Maroofian, M. Zamani, T. Seifi, A. Sedaghat,G. Shariati, Y. Jamshidi, H. D. Allen, X. H. T. Wehrens, H. Galehdari, A. P. Landstrom, Analysis of enriched rare variants in JPH2-encoded junctophilin-2 among greater middle eastern individuals reveals a novel homozygous variant associated with neonatal dilated cardiomyopathy, Sci- entific Reports 9 (1).

R. E. Hershberger, A. Morales, J. D. Siegfried, Clinical and genetic issues in dilated cardiomyopathy: A review for genetics professionals, Genetics in Medicine 12 (11) (2010) 655–667.

S. Franciosi, Nexilin: a potential novel factor contributing to dilated car- diomyopathy, Clinical Genetics 77 (6) (2010) 535–536.

J. L. Theis, K. M. Sharpe, M. E. Matsumoto, H. S. Chai, A. A. Nair, J. D. Theis, M. De Andrade, E. D. Wieben, V. V. Michels, T. M. Olson, Homozygosity mapping and exome sequencing reveal gatad1 mutation in autosomal recessive dilated cardiomyopathy, Circulation: Cardiovascular Genetics 4 (6) (2011) 585–594.

R. Knoll, R. Postel, J. Wang, R. Kratzner, G. Hennecke, A. M. Vacaru, P. Vakeel, C. Schubert, K. Murthy, B. K. Rana, D. Kube, G. Knoll, K. Schafer, T. Hayashi, T. Holm, A. Kimura, N. Schork, M. R. Toliat, P. Nurnberg, H.-P. Schultheiss, W. Schaper, J. Schaper, E. Bos, J. D. Her- tog, F. J. van Eeden, P. J. Peters, G. Hasenfuss, K. R. Chien, J. Bakkers, Laminin-$\alpha$4 and integrin-linked kinase mutations cause human cardiomy-opathy via simultaneous defects in cardiomyocytes and endothelial cells, Circulation 116 (5) (2007) 515–525.

T. Arimura, Y. K. Hayashi, T. Murakami, Y. Oya, S. Funabe, E. Arikawa- Hirasawa, N. Hattori, I. Nishino, A. Kimura, Mutational analysis of fukutin gene in dilated cardiomyopathy and hypertrophic cardiomyopathy, Circulation Journal 73 (1) (2009) 158–161.

K. Miyazawa, K. Ito, The evolving story in the genetic analysis for heart failure, Frontiers in Cardiovascular Medicine 8 (2021) 646816.

R. L. Prentice, S. Paczesny, A. Aragaki, L. M. Amon, L. Chen, S. J. Pitteri, M. McIntosh, P. Wang, T. Buson Busald, J. Hsia, et al., Novel proteins associated with risk for coronary heart disease or stroke among postmenopausal women identified by in-depth plasma proteome profiling, Genome medicine 2 (2010) 1–13.

L. Wallentin, N. Eriksson, M. Olszowka, T. B. Grammer, E. Hagström, C. Held, M. E. Kleber, W. Koenig, W. März, R. A. Stewart, et al., Plasma proteins associated with cardiovascular death in patients with chronic coronary heart disease: A retrospective study, PLoS medicine 18 (1) (2021) e1003513.

# Ischemic Heart Disease:

N. G. Frangogiannis, Pathophysiology of myocardial infarction (Sep. 2015).

M. Ambroziak, A. Kury-lowicz, A. Budaj, Increased coagulation factor XIII activity but not genetic variants of coagulation factors is associated with myocardial infarction in young patients, Journal of Thrombosis and Thrombolysis 48 (3) (2019) 519–527.

L. Lu, M. Liu, R. Sun, Y. Zheng, P. Zhang, Myocardial infarction: Symp- toms and treatments, Cell Biochemistry and Biophysics 72 (3) (2015) 865–867.

M. Puzianowska-Ku´znicka, ESR1 in myocardial infarction, Clinica Chim- ica Acta 413 (1-2) (2012) 81–87.

M. Tatsuguchi, M. Furutani, J. ichi Hinagata, T. Tanaka, Y. Furutani, S. ichiro Imamura, M. Kawana, T. Masaki, H. Kasanuki, T. Sawamura, R. Matsuoka, Oxidized LDL receptor gene (OLR1) is associated with the risk of myocardial infarction, Biochemical and Biophysical Research Com- munications 303 (1) (2003) 247–250.

S. ichi Koide, K. Kugiyama, S. Sugiyama, S. ichi Nakamura, H. Fukushima, O. Honda, M. Yoshimura, H. Ogawa, Association of poly- morphism in glutamate-cysteine ligase catalytic subunit gene with coro- nary vasomotor dysfunction and myocardial infarction, Journal of the American College of Cardiology 41 (4) (2003) 539–545.

K. Ozaki, Y. Ohnishi, A. Iida, A. Sekine, R. Yamada, T. Tsunoda, H. Sato, H. Sato, M. Hori, Y. Nakamura, T. Tanaka, Functional SNPs in the lymphotoxin-$\alpha$ gene that are associated with susceptibility to myocardial infarction, Nature Genetics 32 (4) (2002) 650–654.

X. Wang, M. Ria, P. M. Kelmenson, P. Eriksson, D. C. Higgins, A. Sam- negård, C. Petros, J. Rollins, A. M. Bennet, B. Wiman, U. de Faire, C. Wennberg, P. G. Olsson, N. Ishii, K. Sugamura, A. Hamsten, K. Forsman-Semb, J. Lagercrantz, B. Paigen, Positional identification of TNFSF4, encoding OX40 ligand, as a gene that influences atherosclerosis susceptibility, Nature Genetics 37 (4) (2005) 365–372.

K. Ozaki, H. Sato, A. Iida, H. Mizuno, T. Nakamura, Y. Miyamoto, A. Takahashi, T. Tsunoda, S. Ikegawa, N. Kamatani, M. Hori, Y. Naka- mura, T. Tanaka, A functional SNP in PSMA6 confers risk of myocardial infarction in the japanese population, Nature Genetics 38 (8) (2006) 921–925.

K. Thygesen, , J. S. Alpert, A. S. Jaffe, M. L. Simoons, B. R. Chaitman, H. D. White, Third universal definition of myocardial infarction, Nature Reviews Cardiology 9 (11) (2012) 620–633.

D. Girelli, C. Russo, P. Ferraresi, O. Olivieri, M. Pinotti, S. Friso, F. Man- zato, A. Mazzucco, F. Bernardi, R. Corrocher, Polymorphisms in the fac- tor VII gene and the risk of myocardial infarction in patients with coronary artery disease, New England Journal of Medicine 343 (11) (2000) 774–780.

S. Ye, P. Eriksson, A. Hamsten, M. Kurkinen, S. E. Humphries, A. M. Henney, Progression of coronary atherosclerosis is associated with a com- mon genetic variant of the human stromelysin-1 promoter which results in reduced gene expression, Journal of Biological Chemistry 271 (22) (1996) 13055–13060.

L. Wang, E. R. Hauser, S. H. Shah, M. A. Pericak-Vance, C. Haynes, D. Crosslin, M. Harris, S. Nelson, A. B. Hale, C. B. Granger, J. L. Haines, C. J. Jones, D. Crossman, D. Seo, S. G. Gregory, W. E. Kraus, P. J. Goldschmidt-Clermont, J. M. Vance, Peakwide mapping on chromosome 3q13 identifies the kalirin gene as a

novel candidate gene for coronary artery disease, The American Journal of Human Genetics 80 (4) (2007) 650–663.

X. Ma, A common haplotype at the CD36 locus is associated with high free fatty acid levels and increased cardiovascular risk in caucasians, Human Molecular Genetics 13 (19) (2004) 2197–2205.

E. N. Olson, Coronary artery disease and the MEF2a transcription factor, Science of Aging Knowledge Environment 2003 (48).

L. Wang, C. Fan, S. E. Topol, E. J. Topol, Q. Wang, Mutation of MEF2a in an inherited disorder with features of coronary artery disease, Science 302 (5650) (2003) 1578–1581.

A. Mani, J. Radhakrishnan, H. Wang, A. Mani, M.-A. Mani, C. Nelson- Williams, K. S. Carew, S. Mane, H. Najmabadi, D. Wu, R. P. Lifton, Mutation in a family with early coronary disease and metabolic risk fac- tors, Science 315 (5816) (2007) 1278–1282.

S. ichi Nakamura, K. Kugiyama, S. Sugiyama, S. Miyamoto, S. ichi Koide, H. Fukushima, O. Honda, M. Yoshimura, H. Ogawa, Polymorphism in the 5′-flanking region of human glutamate-cysteine ligase modifier subunit gene is associated with myocardial infarction, Circulation 105 (25) (2002) 2968–2973.

D. Moatti, Polymorphism in the fractalkine receptor CX3cr1 as a genetic risk factor for coronary artery disease, Blood 97 (7) (2001) 1925–1928.

M. P. Grover, S. Ballouz, K. A. Mohanasundaram, R. A. George, A. Goscinski, T. M. Crowley, C. D. H. Sherman, M. A. Wouters, Novel therapeutics for coronary artery disease from genome-wide association study data, BMC Medical Genomics 8 (S2).

M. Sheikhvatan, M. A. boroumand, M. Behmanesh, S. Ziaee, S. Cheraghee, Integrin beta-3 gene polymorphism and risk for myocardial in- farction in premature coronary disease, Iranian Journal of Biotechnology 17 (2) (2019) 79–88.

M. J. Ghorbani, N. Razmi, S. M. B. Tabei, M. J. Zibaeenezhad, H. R. Goodarzi, A substitution mutation in lrp8 gene is significantly associated with susceptibility to familial myocardial infarction, ARYA Atherosclerosis 16 (6).

F. S. Czepluch, B. Wollnik, G. Hasenfuß, Genetic determinants of heart failure: facts and numbers (2018).

G. Durmus, N. Karakus, S. Yuksel, N. Kara, Analysis of twelve cardiovascular disease related gene mutations among turkish patients with coronary artery disease, Int. J. Blood Res. Disord 7 (2020) 047.

A. V. Khera, S. Kathiresan, Genetics of coronary artery disease: discovery, biology and clinical translation, Nature Reviews Genetics 18 (6) (2017) 331–344.

C. M. Schooling, J. Huang, J. Zhao, M. Kwok, S. A. Yeung, S. Lin, Dis- connect between genes associated with ischemic heart disease and targets of ischemic heart disease treatments, EBioMedicine 28 (2018) 311–315.

R. Elosua, S. Sayols-Baixeras, The genetics of ischemic heart disease: From current knowledge to clinical implications, Revista Española de Car- diolog´ıa (English Edition) 70 (9) (2017) 754–762.

# Myocardial Fibrosis:


C. Riehle, J. Bauersachs, Of mice and men: models and mechanisms of diabetic cardiomyopathy, Basic Research in Cardiology 114 (1).

I. Falcão-Pires, A. F. Leite-Moreira, Diabetic cardiomyopathy: under- standing the molecular and cellular basis to progress in diagnosis and treatment, Heart Failure Reviews 17 (3) (2011) 325–344.

M. Joshi, S. R. Kotha, S. Malireddy, V. Selvaraju, A. R. Satoskar, A. Palesty, D. W. McFadden, N. L. Parinandi, N. Maulik, Conundrum of pathogenesis of diabetic cardiomyopathy: role of vascular endothelial dysfunction, reactive oxygen species, and mitochondria, Molecular and Cellular Biochemistry 386 (1-2) (2013) 233–249.

U. Varma, P. Koutsifeli, V. Benson, K. Mellor, L. Delbridge, Molecular mechanisms of cardiac pathology in diabetes – experimental in- sights, Biochimica et Biophysica Acta (BBA) - Molecular Basis of Disease 1864 (5) (2018) 1949–1959.

S. Murarka, M. R. Movahed, Diabetic cardiomyopathy, Journal of Cardiac Failure 16 (12) (2010) 971–979.

V. K. Randhawa, S. Dhanvantari, K. A. Connelly, How diabetes and heart failure modulate each other and condition management, Canadian Journal of Cardiology 37 (4) (2021) 595–608.

J. Asbun, F. J. Villarreal, The pathogenesis of myocardial fibrosis in the setting of diabetic cardiomyopathy, Journal of the American College of Cardiology 47 (4) (2006) 693–700.

C. H. Mandavia, A. R. Aroor, V. G. DeMarco, J. R. Sowers, Molecular and metabolic mechanisms of cardiac dysfunction in diabetes, Life Sciences 92 (11) (2013) 601–608.

K. Huynh, B. C. Bernardo, J. R. McMullen, R. H. Ritchie, Diabetic car- diomyopathy: Mechanisms and new treatment strategies targeting antiox- idant signaling pathways, Pharmacology & Therapeutics 142 (3) (2014) 375–415.

X. Palomer, J. Pizarro-Delgado, M. Vázquez-Carrera, Emerging actors in diabetic cardiomyopathy: Heartbreaker biomarkers or therapeutic tar- gets?, Trends in Pharmacological Sciences 39 (5) (2018) 452–467.

A. P. Landstrom, M. S. Parvatiyar, J. R. Pinto, M. L. Marquardt, J. M. Bos, D. J. Tester, S. R. Ommen, J. D. Potter, M. J. Ackerman, Molecular and functional characterization of novel hypertrophic cardiomyopathy susceptibility mutations in TNNC1-encoded troponin c, Journal of Molecular and Cellular Cardiology 45 (2) (2008) 281–288.

A. Marian, Pathogenesis of diverse clinical and pathological phenotypes in hypertrophic cardiomyopathy, The Lancet 355 (9197) (2000) 58–60.

M. Brownlee, Biochemistry and molecular cell biology of diabetic complications, Nature 414 (6865) (2001) 813–820.

G. Jia, V. G. DeMarco, J. R. Sowers, Insulin resistance and hyperinsulinemia in diabetic cardiomyopathy, Nature Reviews Endocrinology 12 (3) (2015) 144–153.

M. Knapp, X. Tu, R. Wu, Vascular endothelial dysfunction, a major mediator in diabetic cardiomyopathy, Acta Pharmacologica Sinica 40 (1) (2018) 1–8.

Y. Tan, Z. Zhang, C. Zheng, K. A. Wintergerst, B. B. Keller, L. Cai, Mechanisms of diabetic cardiomyopathy and potential therapeutic strate- gies: preclinical and clinical evidence, Nature Reviews Cardiology 17 (9) (2020) 585–607.

P. Sorajja, The molecular genetics of hypertrophic cardiomyopathy: prognostic implications, Europace 2 (1) (2000) 4–14.

S. Morimoto, Sarcomeric proteins and inherited cardiomyopathies, Cardiovascular Research 77 (4) (2007) 659–666.

C.-R. Chong, K. Clarke, E. Levelt, Metabolic remodelling in diabetic car- diomyopathy, Cardiovascular Research 113 (4) (2017) 422–430.

F. Cambronero, F. Marin, V. Roldan, D. Hernandez-Romero, M. Valdes, G. Y. Lip, Biomarkers of pathophysiology in hypertrophic cardiomyopa- thy: implications for clinical management and prognosis, European Heart Journal 30 (2) (2008) 139–151.

E. Blair, Mutations in the gamma2 subunit of AMP-activated protein kinase cause familial hypertrophic cardiomyopathy: evidence for the central role of energy compromise in disease pathogenesis, Human Molecular Genetics 10 (11) (2001) 1215–1220.

C. M. Thomas, Q. C. Yong, R. M. Rosa, R. Seqqat, S. Gopal, D. E. Casarini, W. K. Jones, S. Gupta, K. M. Baker, R. Kumar, Cardiac-specific suppression of NF-$\kappa$b signaling prevents diabetic cardiomyopathy via inhibition of the renin-angiotensin system, American Journal of Physiology- Heart and Circulatory Physiology 307 (7) (2014) H1036–H1045.

V. P. Singh, K. M. Baker, R. Kumar, Activation of the intracellular renin- angiotensin system in cardiac fibroblasts by high glucose: role in extra- cellular matrix production, American Journal of Physiology-Heart and Circulatory Physiology 294 (4) (2008) H1675–H1684.

J. a Kim, Y. Wei, J. R. Sowers, Role of mitochondrial dysfunction in insulin resistance, Circulation Research 102 (4) (2008) 401–414.

S. Boudina, E. D. Abel, Diabetic cardiomyopathy revisited, Circulation 115 (25) (2007) 3213–3223.

A. Lorenzo-Almorós, J. Tuñón, M. Orejas, M. Cort´es, J. Egido, Ó . Lorenzo, Diagnostic approaches for diabetic cardiomyopathy, Cardio- vascular Diabetology 16 (1).

V. Ormazabal, S. Nair, O. Elfeky, C. Aguayo, C. Salomon, F. A. Zuñiga, Association between insulin resistance and the development of cardiovas- cular disease, Cardiovascular Diabetology 17 (1).

J. Gollmer, A. Zirlik, H. Bugger, Established and emerging mechanisms of diabetic cardiomyopathy, Journal of Lipid and Atherosclerosis 8 (1) (2019) 26.

M. R. Taylor, E. Carniel, L. Mestroni, Familial hypertrophic cardiomy- opathy: clinical features, molecular genetics and molecular genetic testing, Expert Review of Molecular Diagnostics 4 (1) (2004) 99–113.

V. P. Singh, B. Le, R. Khode, K. M. Baker, R. Kumar, Intracellular angiotensin II production in diabetic rats is correlated with cardiomyocyte apoptosis, oxidative stress, and cardiac fibrosis, Diabetes 57 (12) (2008) 3297–3306.

P. K. Mishra, W. Ying, S. S. Nandi, G. K. Bandyopadhyay, K. K. Patel, S. K. Mahata, Diabetic cardiomyopathy: An immunometabolic perspective, Frontiers in Endocrinology 8.

F. Westermeier, J. A. Riquelme, M. Pavez, V. Garrido, A. D´ıaz, H. E. Verdejo, P. F. Castro, L. Garc´ıa, S. Lavandero, New molecular insights of insulin in diabetic cardiomyopathy, Frontiers in Physiology 7.

M. Zamora, J. A. Villena, Contribution of impaired insulin signaling to the pathogenesis of diabetic cardiomyopathy, International Journal of Molec- ular Sciences 20 (11) (2019) 2833.

I. Evangelista, R. Nuti, T. Picchioni, F. Dotta, A. Palazzuoli, Molecu- lar dysfunction and phenotypic derangement in diabetic cardiomyopathy, International Journal of Molecular Sciences 20 (13) (2019) 3264.

S. P. Levick, A. Widiapradja, The diabetic cardiac fibroblast: Mechanisms underlying phenotype and function, International Journal of Molecular Sciences 21 (3) (2020) 970.

M. K. Brahma, M. E. Pepin, A. R. Wende, My sweetheart is broken: Role of glucose in diabetic cardiomyopathy, Diabetes & Metabolism Journal 41 (1) (2017) 1.

L. Athithan, G. S. Gulsin, G. P. McCann, E. Levelt, Diabetic cardiomy- opathy: Pathophysiology, theories and evidence to date, World Journal of Diabetes 10 (10) (2019) 490–510.

S. Chandra, K. C. Ehrlich, M. Lacey, C. Baribault, M. Ehrlich, Epigenetics and expression of key genes associated with cardiac fibrosis: Nlrp3, mmp2, mmp9, ccn2/ctgf and agt, Epigenomics 13 (03) (2021) 219–234.

C. Hartmann, A. F. R. D. S. Miggiolaro, J. d. S. J. Motta, L. Baena Carstens, C. Busatta Vaz De Paula, S. Fagundes Grobe, L. Her- mann de Souza Nunes, G. Lenci Marques, P. Libby, L. Zytynski Moura, et al., The pathogenesis of covid-19 myocardial injury: an immunohistochemical study of postmortem biopsies, Frontiers in immunology (2021) 4624.

J. Zhao, T. Lv, J. Quan, W. Zhao, J. Song, Z. Li, H. Lei, W. Huang, L. Ran, Identification of target genes in cardiomyopathy with fibrosis and cardiac remodeling, Journal of biomedical science 25 (2018) 1–10.

U. Tayal, S. Prasad, S. A. Cook, Genetics and genomics of dilated cardiomyopathy and systolic heart failure, Genome medicine 9 (1) (2017) 1–14.

# Post Viral Myocarditis:


J. M. Lapp´e, C. M. Pelfrey, W. W. Tang, Recent insights into the role of autoimmunity in idiopathic dilated cardiomyopathy, Journal of Cardiac Failure 14 (6) (2008) 521–530.

J. Mason, Myocarditis and dilated cardiomyopathy an inflammatory link, Cardiovascular Research 60 (1) (2003) 5–10.

W.-M. Franz, O. J. Müller, H. A. Katus, Cardiomyopathies: from genetics to the prospect of treatment, The Lancet 358 (9293) (2001) 1627–1637.

J. A. Towbin, N. E. Bowles, The failing heart, Nature 415 (6868) (2002) 227–233.

P. E. Tam, Coxsackievirus myocarditis: Interplay between virus and host in the pathogenesis of heart disease, Viral Immunology 19 (2) (2006) 133–146.

A. Luk, E. Ahn, G. S. Soor, J. Butany, Dilated cardiomyopathy: a review, Journal of Clinical Pathology 62 (3) (2008) 219–225.

D. Fatkin, R. M. Graham, Molecular mechanisms of inherited cardiomyopathies, Physiological Reviews 82 (4) (2002) 945–980.

P. P. Liu, J. W. Mason, Advances in the understanding of myocarditis, Circulation 104 (9) (2001) 1076–1082.

R. Jahns, V. Boivin, L. Hein, S. Triebel, C. E. Angermann, G. Ertl, M. J. Lohse, Direct evidence for a $\beta$1-adrenergic receptor–directed autoimmune attack as a cause of idiopathic dilated cardiomyopathy, Journal of Clinical Investigation 113 (10) (2004) 1419–1429.

P. Zhao, Pathogenesis and therapy of autoimmunity-induced dilated cardiomyopathy, Frontiers in Bioscience Volume (14) (2009) 1708.

K. U. Knowlton, CVB infection and mechanisms of viral cardiomyopathy, in: Current Topics in Microbiology and Immunology, Springer Berlin Heidelberg, pp. 315–335.

S. Pankuweit, I. Portig, B. Maisch, Pathophysiology of cardiac inflammation: Molecular mechanisms, Herz 27 (7) (2002) 669–676.

C. Badorff, G.-H. Lee, K. U. Knowlton, Enteroviral cardiomyopathy: Bad news for the dystrophin-glycoprotein complex, Herz 25 (3) (2000) 227–232.

C. Badorff, K. U. Knowlton, Dystrophin disruption in enterovirus-induced myocarditis and dilated cardiomyopathy: from bench to bedside, Medical Microbiology and Immunology 193 (2-3) (2004) 121–126.

H. S. Li, D. L. Ligons, N. R. Rose, Genetic complexity of autoimmune myocarditis, Autoimmunity Reviews 7 (3) (2008) 168–173.

C. B. Coyne, J. M. Bergelson, Virus-induced abl and fyn kinase signals permit coxsackievirus entry through epithelial tight junctions, Cell 124 (1) (2006) 119–131.

C. Fairley, M. Ryan, P. Wall, J. Weinberg, The organisms reported to cause myocarditis and pericarditis in england and wales, Journal of Infec- tion 32 (3) (1996) 223–225.

L. T. Cooper, Myocarditis, New England Journal of Medicine 360 (15) (2009) 1526–1538.

I. Portig, A. Sandmoeller, S. Kreilinger, B. Maisch, Hla-dqb1* polymor- phism and associations with dilated cardiomyopathy, inflammatory dilated cardiomyopathy and myocarditis, Autoimmunity 42 (1) (2009) 33–40.

R. Dennert, H. J. Crijns, S. Heymans, Acute viral myocarditis, European Heart Journal 29 (17) (2008) 2073–2082.

G. Gao, H. Luo, The ubiquitin–proteasome pathway in viral infections This paper is one of a selection of papers published in this special issue, entitled young investigator s forum., Canadian Journal of Physiology and Pharmacology 84 (1) (2006) 5–14.

M. Esfandiarei, B. M. McManus, Molecular biology and pathogenesis of viral myocarditis, Annual Review of Pathology: Mechanisms of Disease 3 (1) (2008) 127–155.

C. Kawai, From myocarditis to cardiomyopathy: Mechanisms of inflammation and cell death, Circulation 99 (8) (1999) 1091–1100.

Y. Maekawa, M. Ouzounian, M. A. Opavsky, P. P. Liu, Connecting the missing link between dilated cardiomyopathy and viral myocarditis, Cir- culation 115 (1) (2007) 5–8.

G. Castellano, F. Affuso, P. D. Conza, S. Fazio, Myocarditis and dilated cardiomyopathy: possible connections and treatments, Journal of Cardio- vascular Medicine 9 (7) (2008) 666–671.

G. Fung, H. Luo, Y. Qiu, D. Yang, B. McManus, Myocarditis, Circulation research 118 (3) (2016) 496–514.

C. Tschöpe, E. Ammirati, B. Bozkurt, A. L. Caforio, L. T. Cooper, S. B. Felix, J. M. Hare, B. Heidecker, S. Heymans, N. Hübner, et al., My- ocarditis and inflammatory cardiomyopathy: current evidence and future directions, Nature reviews cardiology 18 (3) (2021) 169–193.

A. Cannata', J. Artico, P. Gentile, M. Merlo, G. Sinagra, Myocarditis evolving in cardiomyopathy: when genetics and offending causes work together, European Heart Journal Supplements 21 (Supplement B) (2019) B90–B95.

# Deep Vein Thrombosis:

E. A. BASTOUNIS, A. J. KARAYIANNAKIS, G. G. MAKRI, D. ALEXIOU, E. L. PAPALAMBROS, The incidence of occult cancer in patients with deep venous thrombosis: a prospective study, Journal of Internal Medicine 239 (2) (1996) 153–156.

Y. Peng, T. Wang, Y. Zheng, A. Lian, D. Zhang, Z. Xiong, Z. Hu, K. Xia, C. Shu, A novel variation of SERPINC1 caused deep venous thrombosis in a chinese family, Medicine 98 (1) (2019) e13999.

M. D. Do, D. V. Pham, L. P. Le, L. H. Gia Le, L. B. Minh Tran, M. D. Dang Huynh, Q. M. Do, H. A. Vu, N. H. Nguyen, T. P. Mai, Recurrent proc and novel pros1 mutations in vietnamese patients diagnosed with idiopathic deep venous thrombosis, International journal of laboratory hematology 43 (2) (2021) 266–272.

F. ROSENDAAL, P. REITSMA, Genetics of venous thrombosis, Journal of Thrombosis and Haemostasis 7 (2009) 301–304.

D. Scarvelis, P. S. Wells, Diagnosis and treatment of deep-vein thrombosis, Canadian Medical Association Journal 175 (9) (2006) 1087–1092.

H.-t. Lan, Z.-j. Tong, Y. Ma, H.-t. Han, M. Zhong, Z.-h. Wang, Inte- grated bioinformatics analysis identifies microrna-200a-5p as a new plasma marker in patients with venous thromboembolism, Annals of Vascular Surgery 84 (2022) 354–370.

Z. Jiang, J. Ma, Q. Wang, F. Wu, J. Ping, L. Ming, Circulating microrna expression and their target genes in deep vein thrombosis: a systematic review and bioinformatics analysis, Medicine 96 (50).

L. A. Lotta, M. Wang, J. Yu, I. Martinelli, F. Yu, S. M. Passamonti, D. Consonni, E. Pappalardo, M. Menegatti, S. E. Scherer, et al., Identifica- tion of genetic risk variants for deep vein thrombosis by multiplexed next- generation sequencing of 186 hemostatic/pro-inflammatory genes, BMC Medical Genomics 5 (2012) 1–12.

I. D. Bezemer, L. A. Bare, C. J. Doggen, A. R. Arellano, C. Tong, C. M. Rowland, J. Catanese, B. A. Young, P. H. Reitsma, J. J. Devlin, et al., Gene variants associated with deep vein thrombosis, Jama 299 (11) (2008) 1306–1314.

R. A. Baylis, N. L. Smith, D. Klarin, E. Fukaya, Epidemiology and genetics of venous thromboembolism and chronic venous disease, Circulation research 128 (12) (2021) 1988–2002.
